# Exploring Multifunctional Markers of Biological Age in Farmed Gilthead Sea Bream (*Sparus aurata*): A Transcriptomic and Epigenetic Interplay for an Improved Fish Welfare Assessment Approach

**DOI:** 10.3390/ijms25189836

**Published:** 2024-09-11

**Authors:** Álvaro Belenguer, Fernando Naya-Català, Josep Àlvar Calduch-Giner, Jaume Pérez-Sánchez

**Affiliations:** Instituto de Acuicultura Torre de la Sal (IATS, CSIC), 12595 Ribera de Cabanes, Castellón, Spain; a.belenguer@csic.es (Á.B.); fernando.naya@iats.csic.es (F.N.-C.); j.calduch@csic.es (J.À.C.-G.)

**Keywords:** biological age, chronological age, DNA methylation, epigenetics, transcriptomics, fish welfare

## Abstract

DNA methylation clocks provide information not only about chronological but also biological age, offering a high-resolution and precise understanding of age-related pathology and physiology. Attempts based on transcriptomic and epigenetic approaches arise as integrative biomarkers linking the quantification of stress responses with specific fitness traits and may help identify biological age markers, which are also considered welfare indicators. In gilthead sea bream, targeted gene expression and DNA methylation analyses in white skeletal muscle proved *sirt1* as a reliable marker of age-mediated changes in energy metabolism. To complete the list of welfare auditing biomarkers, wide analyses of gene expression and DNA methylation in one- and three-year-old fish were combined. After discriminant analysis, 668 differentially expressed transcripts were matched with those containing differentially methylated (DM) regions (14,366), and 172 were overlapping. Through enrichment analyses and selection, two sets of genes were retained: 33 showing an opposite trend for DNA methylation and expression, and 57 down-regulated and hypo-methylated. The first set displayed an apparently more reproducible and reliable pattern and 10 multifunctional genes with DM CpG in regulatory regions (*sirt1*, *smad1*, *ramp1*, *psmd2*—up-regulated; *col5a1*, *calcrl*, *bmp1*, *thrb*, *spred2*, *atp1a2*—down-regulated) were deemed candidate biological age markers for improved welfare auditing in gilthead sea bream.

## 1. Introduction

The aquaculture industry is rapidly expanding and becoming more intensified to meet the increased global demand for fish protein [1,2]. This intensification of production may bring about impairments in health and growth performance [3,4,5], which has contributed to the increase in ethical concerns among consumers regarding aquaculture welfare [6,7]. As such, it is essential that the monitoring of welfare status covers a wide range of welfare indicators, either input based, referring to the conditions in which the animals are subjected, or outcome based, referring to the degree of fulfilment of their welfare needs [8,9,10]. Examples of the first type of indicators are stocking density or water oxygen concentration and temperature, whereas growth performance, survival rates, external appearance (skin/fin erosion), sex ratio or maturation state are outcome-based indicators. Another approach for classifying welfare indicators is to divide them by whether they are practical and easy to use on a farm (operational welfare indicators, OWIs), or complex and difficult without access to a laboratory for evaluation (laboratory welfare indicators, LABWIs). However, the range and diversity of welfare needs are constantly evolving as more knowledge and new technologies become available. As such, it is noteworthy that common measurements of cortisol on blood plasma are now moving towards other target tissues (e.g., feces, scales and mucus) [11] to minimize large variability and dramatic increases by the sampling itself in the aquatic environment [12,13], though each target tissue would provide different information on the welfare state and particularly on the timing of the stress response. Likewise, behavioral monitoring is often difficult in the aquatic environment [14,15], but the use of miniaturized data-loggers externally attached to the operculum of sentinel fish represents a practical solution for continuous and accurate individual tracking of swimming activity and breathing rates in tank-based rearing systems [16].

The monitoring of the microorganisms living in or around the farmed animals is also a very promising aquaculture welfare indicator that can serve to plan corrective actions to mitigate an environmental issue, or to modulate the physiological state and the response of the organism from a holobiont perspective [17]. Thus, experimental evidence supports the use of some bacteria taxa as strong markers of thermal stress [18,19] or microplastic exposure [20,21] in both salmonid and non-salmonid fish, which could be monitored in a real-time and cost-effective manner with the advent of the 16S metabarcoding techniques based on Nanopore technology [22,23], though we are far from establishing references values for a wide spectrum of species and culture conditions across the production cycle [24,25]. Other welfare indicators that can aid in developing an improved welfare assessment system are those based on age-related cellular and molecular modifications. Certainly, age-associated DNA methylation changes allow the construction of highly accurate age estimators, termed “epigenetic” or “DNA methylation” clocks, which have been developed for a number of vertebrates, including mammals, birds and fish [26,27,28]. Thus, several piscine DNA methylation clocks have been built to be applied in fisheries and conservation biology programs, with a focus on searching CpGs that mostly reflect the chronological age [28]. Nevertheless, many age-related epigenetic variations are also sensitive to environmental influences [29], either in mammals [30] or fish [31], and there is accumulating evidence that, depending on the CpG loci used, epigenetic clocks can inform not only about chronological but also biological age [32,33,34], which brings the possibility of a high-resolution and precise understanding of the age-related pathology and physiology of an individual [35,36]. 

According to the above findings, epigenetic markers of biological age have been proposed as reliable markers of cumulative welfare in animal production [37,38,39,40]. However, the integration of data from more than one omics layer will add potential value to the measure [34,41], and the combination of transcriptomic and DNA methylation data may link in a more precise manner age-regulated epigenetic marks with specific cellular functions or fitness traits [41,42]. This also applies to fish, and such an integrative omic approach has been used in gilthead sea bream to evaluate the success of nutritional programming in the offspring of broodstock fish fed low fish meal/fish oil diets [43], to reveal interactions between genotype and temperature in male sex determination and spermatogenesis in zebrafish [44], or to better understand the challenge of moderate hypoxia and temperature increases in Atlantic salmon [45]. Likewise, a targeted transcriptomic/epigenetic approach has disclosed the use of the changing gene expression and DNA methylation pattern of sirtuin 1 as a marker of age- and seasonally mediated changes in energy metabolism in the skeletal muscle of gilthead sea bream, whereas this gene was not a good marker in other metabolically active tissues, such as the liver [46]. However, to the best of our knowledge, the integration of wide transcriptomic and DNA methylation data as markers of biological age remains to be largely explored in farmed fish, though experimental evidence highly supported a strong effect of main aquaculture stressors on several biological functions [47,48] through epigenetic marks preferentially located at the gene-promoter regulatory regions [49,50,51]. In agreement with all this, our starting hypothesis was that the integration of wide transcriptomic and epigenomic approaches, filtered by the involvement in multiple functions and differentially methylated CpGs in the gene-promoter regulatory region, would provide a list of putative gene markers of biological age for an extended and precise assessment of cumulative fish welfare. For that purpose, massive gene expression analysis (RNA-seq) combined with a genome-wide DNA methylation (MBD-seq) approach was conducted to disclose the different omics multilayer of white skeletal muscle in one- and three-year-old gilthead sea bream, the most highly cultured fish in the Mediterranean region. This enabled identifying up to 10 age-regulated genes, with particular epigenetic marks and multifunction capabilities, as an array of potential biological age gene markers that will require further validation in independent studies.

## 2. Results

### 2.1. Age-Related Patterns of Gene Expression and Their Discriminant Analysis

**Figure 1:**
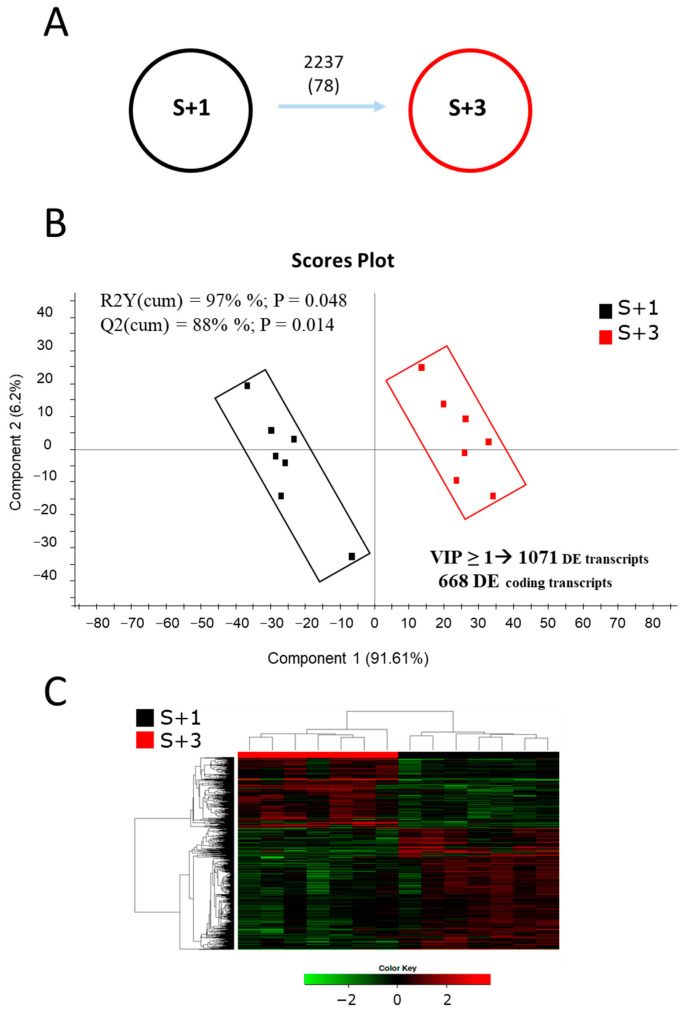
(**A**) Differentially expressed (DE) transcripts between one- (S + 1) and three-year-old fish (S + 3). Numbers indicate DE transcripts (based on the Wald test *p* < 0.05 or FDR-adjusted *p* < 0.05, in brackets). (**B**) Scores plot of partial least-squares discriminant analysis (PLS-DA) of muscular transcripts from S + 1 and S + 3 animals. In the analysis, RNA-seq data were normalized values of differentially expressed transcripts (Wald test, *p* < 0.05). (**C**) Heatmap showing the abundance distribution (z-score) of the DE genes identified to be responsible for the separation between age groups.

Illumina sequencing of mRNA muscle samples from three- (S + 3) and one- (S + 1) year-old animals generated ~526 million single-end (SE) reads (1 × 75), with an average of ~37.6 million reads per sample (Table S1). After trimming and quality filtering, approximately 0.4% of all muscle reads were removed, and the remaining reads ranged between 30.2 million (~2.27 Gb) and 46.9 million (~3.52 Gb) in all samples. Over 90% of these pre-processed reads were mapped against the IATS-CSIC gilthead sea bream reference genome, which retrieved 43,257 expressed coding transcripts (78% of total predicted unique transcripts and corresponding to 20,764 unique descriptions, UD) and 52,844 non-coding transcripts. Differential gene expression analysis between S + 3 and S + 1 fish resulted in 2237 transcripts significantly changing between groups when the Wald test (*p* < 0.05) was applied (Figure 1A), whereas the number of differentially expressed (DE) transcripts decreased to 78 with FDR-adjusted *p* < 0.05. To better explore the differences due to age, a partial least squares-discriminant analysis (PLS-DA) was performed with the data filtered by DE (*p* < 0.05). A clear separation of both groups was observed (Figure 1B), with segregation being mainly due to component 1 (91.6%), in a discriminant model that was validated significantly (explained variance, R2Y = 97%, and predicted variance, Q2 = 88%). Over 1000 discriminant transcripts (VIP ≥ 1) were identified, with 668 known coding transcripts that were generally down-regulated (75.3%) in older fish (S + 3 vs. S + 1). Of note, all animals were correctly classified by hierarchical cluster analysis in each group (Figure 1C). As a validation procedure, eight DE transcripts with age were selected, and their fold-change values calculated using real-time PCR were quite consistent (r = 0.988) with those of the RNA-seq analysis (Table S2). 

### 2.2. Age-Related Patterns of Differentially Methylated DNA and Its Discriminant Analysis

Using MBD-seq, ~568 million SE reads (1 × 75), containing at least one methylated CpG, were obtained, with an average of ~40.5 million reads per sample (Table S1). After trimming and quality filtering, approximately 8% of all muscle reads were removed, and the remaining reads ranged between 30.2 million (~2.27 Gb) and 46.9 million (~3.52 Gb) in all samples. Over 95% of these pre-processed reads were mapped against the IATS-CSIC gilthead sea bream reference genome (1,6 Gb), which was separated into 25 bp windows (the unit employed to calculate the level of methylation). A total of ~10.9M 25 bp genomic regions that potentially contained CG dinucleotides susceptible to being modified by methylation were identified by that analysis. These methylated regions spanned ~273 Mb of the total reference genome, and comprised ~14.1M CpG (77.5% of the total 18.3M CG in the gilthead sea bream genome).

**Figure 2:**
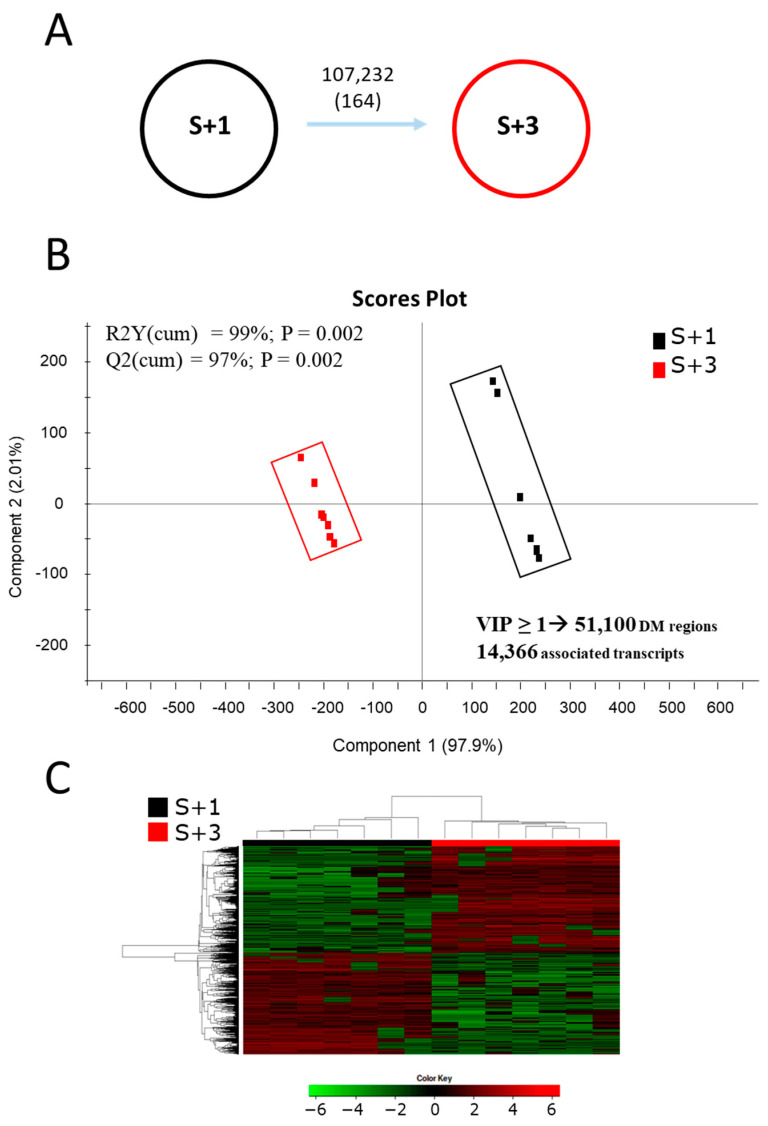
(**A**) Differentially methylated (DM) regions between one- (S + 1) and three-year-old fish (S + 3). Numbers indicate DM regions (based on the *t*-test *p* < 0.05 or FDR-adjusted *p* < 0.05, in brackets). (**B**) Scores plot of partial least-squares discriminant analysis (PLS-DA) of muscular methylated regions from S + 1 and S + 3 animals. MBD-seq data were the normalized values of differentially methylated 25 bp genomic regions (*t*-test, *p* < 0.05). (**C**) Heatmap showing the abundance distribution (z-score) of the DM regions identified to be responsible for the separation between age groups.

Differential methylation studies found a total of 107,232 25 bp genomic regions changing between S + 3 and S + 1 groups when the *t*-test (*p* < 0.05) was applied (Figure 2A), while the number of differentially methylated (DM) regions decreased to 164 with FDR-adjusted *p* < 0.05. The normalized methylation values (rpkm, reads per kilo base per million mapped reads) of those DM regions were used as input in discriminant analysis (PLS-DA) to assess the effect of age over DNA methylation patterns. Like RNA-seq results, the discriminant separation was clear and statistically significant (*p* < 0.01; Figure 2B) when both groups were compared. The segregation was mostly due to component 1 (97.9%) and all animals were again correctly classified by the hierarchical clustering analyses in each group (Figure 2C). The resulting model showed percentage values of explained (R2Y) and predicted (Q2) variance of approximately 99% and 97%, respectively (Figure 2B). After applying a VIP threshold (VIP ≥ 1) in the validated PLS-DA model, up to 51,100 25 bp DM genomic regions were responsible for the separation between experimental groups. These discriminant DM regions appeared to be located in the promoter/coding sequence of 14,366 DM transcripts (Figure 2B), and most of those regions (58.5%) presented a hypo-methylated condition with advancing age (S + 3 vs. S + 1).

### 2.3. Functional Network Analysis of Differentially Expressed Transcripts and of Transcripts with Concomitant Differential Expression and Methylation

**Figure 3:**
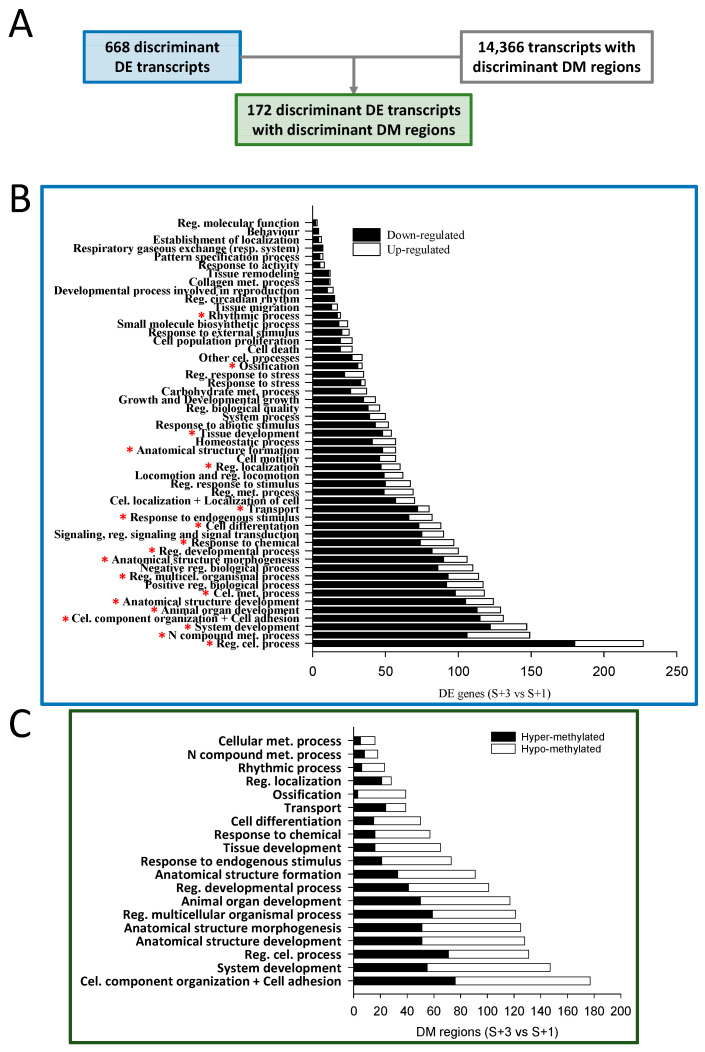
(**A**) Numbers of discriminant DE transcripts, discriminant DM transcripts (containing DM regions), and the overlapping discriminant DE transcripts with DM regions. Bar plots describing the results of an over-representation test conducted over the GO-BP terms of the discriminant DE transcripts (**B**) and of the overlapping discriminant DE transcripts with DM regions (**C**). * indicates that the supra-category is also detected in C. Met., metabolic; Cel., cellular; Reg., regulation; Multicel., multicellular.

When results from S + 3 vs. S + 1 fish were compared and the identified discriminant DE transcripts known to be located in coding areas (668) were matched with those containing DM regions of discriminant value (14,366), only 172 were overlapping (Figure 3A). The over-representation analysis of the list of discriminant DE coding transcripts (668) discerned 442 enriched functions (Gene ontology-Biological process, GO-BP, unique terms; Table S3). The obtained over-represented functions were clustered in 51 supra-categories (GO-BP ancestors), and the divergent numbers of DE transcripts within each one are shown in Figure 3B. The GO-BP ancestor Regulation of cellular process presented the largest number of DE transcripts, followed by N compound metabolic process and System development. Interestingly, the expression of most transcripts in all assigned supra-categories (nearly 80% globally) were down-regulated with age (S + 3 vs. S + 1 animals) and only approximately 20% presented an up-regulated gene expression.

A functional network analysis of the 172 overlapping transcripts (DE containing DM regions) was also conducted, displaying up to 81 different GO-BP terms (Table S3). These over-represented functions were clustered in 19 GO-BP ancestors, and the different numbers of DM regions within each one are shown in Figure 3C. Among these regions, approximately 52% presented hypo methylation when S + 3 and S + 1 fish were compared, and the remaining 48% were hyper-methylated. This contrasts with the proportions of down- and up-regulated DE transcripts after the enrichment analysis. When the results of the latter analysis (Figure 3B) were compared with those of the enrichment of the overlapping DE transcripts with DM regions (Figure 3C), all GO-BP ancestors identified in the latter appeared in the former, containing most of them a substantial number of DE transcripts.

### 2.4. The Different Profiles of DNA Methylation and Expression

**Figure 4:**
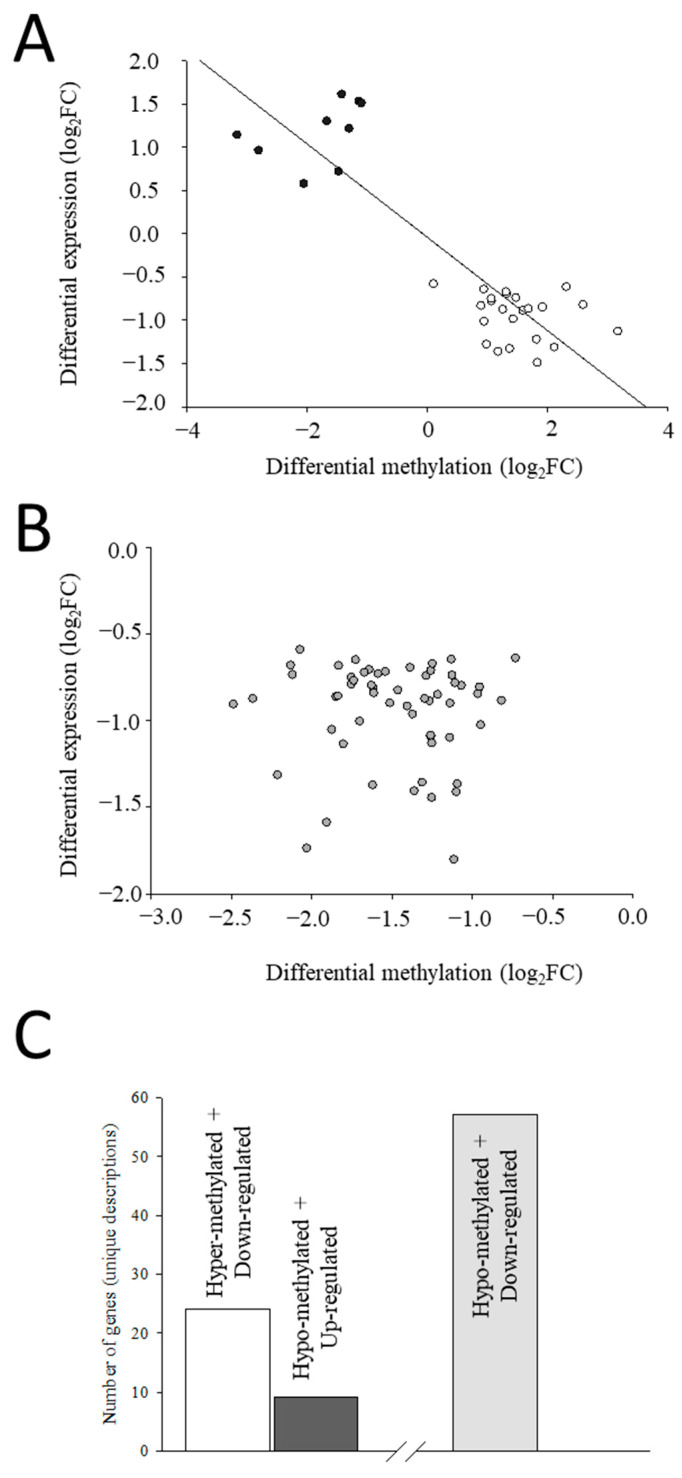
Correlation analysis between differentially methylation and differential expression (both as log2 fold change in rpkm values between S + 3 and S + 1 fish) of the overlapping transcripts (dots in figure) showing an opposite effect of DNA methylation and expression (**A**) and of the down-regulated overlapping transcripts associated to hypo-methylated CpGs (**B**). (**C**) Bar plot representing the numbers of selected genes showing an opposite trend for DNA methylation and expression (hyper-methylated + down-regulated and hypo-methylated + up-regulated).

After the enrichment analysis of the 172 overlapping transcripts, the resulting 108 enriched transcripts were identified and separated in two groups. First, we selected those transcripts with DM regions presenting an opposite trend for DNA methylation and expression (i.e., hypo-methylated regions with up-regulation of the matching transcript or vice versa). When transcripts that contained at least 75% of their DM regions with a negative correlation were filtered, a total of 33 transcripts (33 UD), corresponding to 141 regions, were displayed, and they presented a strong and significant negative relationship between differential methylation and expression (Figure 4A). Regarding in-depth these genes, the location of the DM regions and the number of CpGs are shown in Table S4. Additionally, the genomic organization and number of position of DM CpGs are graphically represented in Figure S1.

The second group contained down-regulated transcripts containing hypo-methylated regions and, again, they were filtered for those with at least 75% of their DM regions with hypo methylation. A total of 61 transcripts (57 UD), containing 140 DM regions, were identified. In this case, there was no significant relationship within this group of transcripts between differential methylation and expression (Figure 4B), which indicates that, unlike the first set of genes, no clear pattern on the impact of differential methylation on expression was observed here. The location of the DM regions and the number of CpGs of the latter transcripts are also presented in Table S5, and their genomic organization and number of position of DM CpGs are graphically represented in Figure S2. Overall, most genes (UD) were down-regulated (24 containing hyper-methylated regions and 57 with hypo-methylated CpGs), and only a few presented an up-regulated expression (9 associated to hypo-methylated regions). Only those from the first group, presenting a clearer relationship between gene expression and level of DNA methylation (opposite trend, 24 hyper-methylated and down-regulated and 9 hypo-methylated and up-regulated, Figure 4C), were retained.

### 2.5. Candidate Markers of Biological Age 

The selected genes had been previously assigned, as mentioned, to one of the 19 GO-BP ancestors through the functional network analysis, and the corresponding genes within each biological function are presented in Table 1 (i.e., down-regulated genes linked with hyper-methylated regions or vice versa). The functional processes are ordered by the number of associated genes, with system development of the GO-BP term having the greatest gene number, followed by anatomical structure development and anatomical structure morphogenesis. Additionally, these selected genes are ordered by the number of associated functions (Table 2 and Table S4), and the first 14 genes, chosen by their involvement in multiple functions (i.e., at least 4 processes), were selected and are presented in Table 2 (*sirt1*, NAD-dependent deacetylase sirtuin-1; *smad1*, Mothers against decapentaplegic homolog 5; *col5a1*, Collagen alpha-1(V) chain; *calcrl*, Calcitonin gene-related peptide type 1 receptor-like; *nrp2*, Neuropilin-2; *bmp1*, Bone morphogenetic protein 1; *ramp1*, Receptor activity-modifying protein 1; *abi3bp*, Target of Nesh-SH3; *mical2*, Protein MICAL-2; *thrb*, thyroid hormone receptor beta; *spred2*, sprouty-related, EVH1 domain-containing protein 2; *arhgap24*, Rho GTPase-activating protein 24; *psmd2*, 26S proteasome non-ATPase regulatory subunit 2; *atp1a2*, sodium/potassium-transporting ATPase subunit alpha-2). Interestingly, among the 14 selected genes, a substantial proportion (71%; 10 out of 14) contained at least one regulatory DM region (i.e., positioned in promoter, the first intron or the first exon). Thus, those 10 genes with a methylation mark in a regulating region (*sirt1*, *smad1*, *ramp1*, *psmd2*—up-regulated; *col5a1*, *calcrl*, *bmp1*, *thrb*, *spred2*, *atp1a2*—down-regulated) have been proposed as putative candidate markers of biological age.

## 3. Discussion

Refining approaches to safeguard and optimize the well-being of fish can help to increase the profitability and sustainability of aquaculture through the production of more robust fish. There is now a vast array of fish welfare indicators, but there is little consensus on the most adequate criteria to evaluate differences in welfare needs among species, rearing systems or biological features [52,53]. Thus, in the gonochoristic teleost European sea bass, elevated temperatures during early development (thermosensitive period) resulted in a masculinization of the population [54,55]. Conversely, an accelerated male–female sex reversal in the protandrous hermaphrodite gilthead sea bream seems to be the phenotypic expression of a cumulative impaired welfare that is both nutritionally and genetically regulated [56,57]. This latter process exemplifies a biological age deviation as the result of a pseudo-feminization process with a lowered estradiol/11ketosterone plasma ratio in reproductive females, which would denote a failure of the negative feedback inhibition of male–female sex reversal. Likewise, at the cellular and molecular level, the present study disclosed a vast number of age-regulated muscle transcripts by comparing one- and three-year-old fish with homogeneous weights within groups and with no sex bias. Indeed, in the hermaphrodite gilthead sea bream nearly all one-year-old fish are usually males and in this work the three-year-old fish employed for sampling had a similar weight of approximately 1 kg, as expected for males at that age compared to the heavier females when fed a control diet with high levels of fish meal and fish oil [57]. Overall, this study finally enabled obtaining an enriched list of 10 candidate markers of biological aging after the integration of two omics layers (transcriptomics and epigenetics) and filtering by multifunctional genes with DM CpGs in regulatory regions, which will require a final functional validation in independent studies.

Age-mediated shifts in gene expression have been reported in numerous species [58,59], though the type of genes that are regulated vary among different tissues and organisms [60]. Accordingly, a clear impact of age on the muscle transcriptome was observed in the current study (Figure 1), which is consistent with previous studies in mammals [61,62] and fish [63,64]. Moreover, we observed that most discriminant coding DE transcripts were down-regulated with advancing age, which would reflect the normal declining with age of a wide range of biological processes [59,65,66], though paradoxically it is not the common feature in a number of previous studies in mammals and fish [61,62,64]. In any case, it is well accepted that aging is related, in mammals [67,68] and fish (killifish) used a model of vertebrate aging, with an overall hypo-methylation that becomes first associated to a down-regulation of DNA methyltransferases. In the present study, this fits well with a global hypo methylation (58.5%) among the discriminant DM regions in our age model of one- and three-year-old fish, that at the same time would co-exist with a site-specific hypermethylation, as reported upon aging in both humans [69] and other animal models [67,70], including fish [28,71,72].

Methylation of DNA is normally associated with the silencing of gene expression [73]. Nonetheless, as mentioned before, most DE genes presented a lowered expression with age regardless of methylation pattern. Certainly, two different types of transcriptomic and epigenetic interplays were found herein with the age-related down-regulated gene expression. Accordingly, a first set of transcripts presented an opposite trend for DNA methylation and expression (33 transcripts and UD), which means that they showed a down-regulated expression due to hyper methylation (24 transcripts) or vice versa (9 transcripts), resulting in a significant negative correlation between DNA methylation and their expression level (Figure 4A). Conversely, a second group of 61 down-regulated transcripts (57 UD) were linked to hypo-methylated DM regions. The effect of the differential methylation on gene expression may rely on the position of the epigenetic mark because increased methylated CpG sites in the promoter region of a gene results normally in shutdown of its expression [74,75,76]. Likewise, methylation of the first exon/intron may be also strongly related to the transcriptional silencing [77,78]. Nevertheless, DNA methylation patterns are far more complex than initially thought. For instance, hypo methylation does not seem to be always an activating epigenetic change, since the loss of DNA methylation in the gene body may be accompanied by formation of repressive chromatin, resulting in a decreased expression [79]. Alternatively, a lower methylation level at gene bodies may result in gene silencing due to nucleosome destabilization in transcribed regions and lower efficiencies of transcription elongation or splicing [80,81]. This hypo methylation-induced gene expression shutdown might contribute to a certain degree of instability in the chromatin or nucleosome, which could explain the less clear pattern on the effect of the reduced level of methylation in this second set of retained transcripts (Figure 4B) and suggests that this impact seems to be less reproducible and reliable. Hence, only the transcripts of the first group, whose changes in expression are likely a result of the direct action of enzymes involved in the DNA methylation or demethylation, were kept herein for further selection.

Among the 33 transcripts presenting an opposite trend for DNA methylation and expression, those linked to a greater number of processes (>4 GO-BP ancestors) were retained as a first list of selected markers. Such a filtering approach rendered 14 multiple function genes that would be more likely affected by environmental insults (e.g., increased temperatures and hypoxia), which in turn serves to disclose a set of molecular markers that become potentially highly responsive to aging and common aquaculture stressors [47,48,82,83]. As such, it must be noted that the resulting list of selected genes is largely involved in developmental processes, which agrees with the fact that aging is viewed as an evolutionary conserved developmental process across mammals [84], and vertebrates in general. Moreover, the observation that most of the above selected genes (10 out of 14; 71%) shared a DM region in their promoter regulatory region further supports a physiological role as age-related markers of environmental perturbations [49,50,51], which makes our final set of 10 genes a reliable list of candidate markers of chronological age deviations.

**Figure 5:**
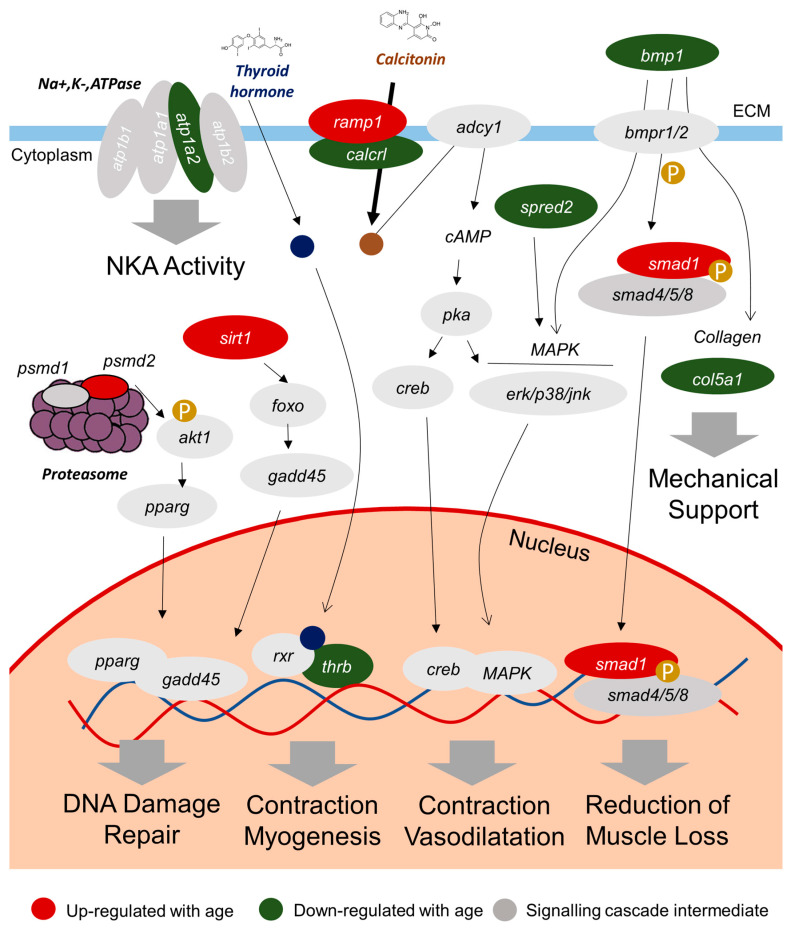
Proposed framework for transduction pathways of the 10 selected biological age markers in the white skeletal muscle of the gilthead sea bream and its mechanisms of methylation. The epigenetically mediated down-regulation of the gene encoding for the Na^+^/K^−^/ATPase subunit *atp1a2* induces a decrease in the NKA activity in old fish and the same pattern for collagen fibers-related *col5a1* provokes a loss of the mechanical support of cells. The hyper methylation of nuclear receptor for thyroid hormone (*thrb*), sprouty-related, EVH1 domain-containing protein 2(*spred2*) and bone morphogenetic protein (*bmp1*) stops the flow of MAPK signaling activation, avoiding advanced-age individuals to propel optimal myogenesis, muscle contraction and vasodilatation. Something similar occurs with calcitonin membrane receptor, in a compensatory mechanism with their components being hyper-methylated (*calcrl*) and hypo-methylated (*ramp1*). Hypo methylation leading to up-regulation is also present in the *smad1* gene, phosphorylated by *bmp1* and probably showing this trend to maximize the deficit on this protein. By last, the *sirt1* and *psmd2* genes are up-regulated by hypo methylation, being probably involved in the modulation of DNA damage repair. Altogether, this induces modulation of the white skeletal muscle activity in advanced-age organisms.

From a functional point of view, a transduction pathway with the proposed mechanisms of the 10 selected genes was shown (Figure 5). The suppression of cellular senescence by SIRTs is mostly mediated through a delay of the age-related telomere attrition, a sustainment of genome integrity, and a promotion of DNA damage repair [85,86,87,88]. Certainly, the increasing evidence of SIRTs in the field of aging and age-related diseases points that they may render novel targets for treating diseases linked with aging and extended human lifespan [89]. This becomes especially evident for SIRT1, the most extensively studied mammalian SIRT. As such, the observation that the gene *sirt1* is included in our final list of biological markers confirms and extends our previous gilthead sea bream study, in which muscle *sirt1* was shaped by age at the transcriptional and epigenetic level in a farmed fish through the production cycle [46]. Moreover, this outcome served as a methodological checkpoint of the procedure that we have used herein for revealing potential markers of chronological age that at the same time have the potential to be regulated at a large extent by a challenging environment. Regarding the gene *psmd2* (26S proteasome non-ATPase regulatory subunit 2), it encodes for one of the non-ATPase subunits of the 19S regulator lid within the 26S proteasome, a multicatalytic proteinase complex that is involved in the ubiquitin–proteasome system, recognized as a major intracellular protein degradation system with an important role for muscle homeostasis and health [90]. It has been indicated that proteasome activity decreases with age in several tissues [91,92], including skeletal muscle [93]. Nevertheless, proteasomes might play a relevant role in DNA damage signaling and DNA repair [94], as previously indicated for SIRTs, and this *psmd2* gene may be implicated in cell cycle and increase cell proliferation [95], which might be related to the high levels of *psmd2* significantly associated with age [95] and explain its up-regulation in our model of older fish.

Other two sets of genes with a complementary role with aging are those of *bmp1*-*smad1* and *ramp1*-*calcrl*. First, *bmp1* (Bone morphogenetic protein 1) encodes for one of the bone morphogenetic proteins (BMP) that attach to dedicated receptors that consecutively phosphorylate BMP-responsive Smad proteins (e.g., Smad1 encoded by gene *smad1*, mothers against decapentaplegic homolog 5) [96]. BMP signaling is essential for the regulation of adult muscle mass in normal and pathological situations [97]. So, it may be speculated that the down-regulation of *bmp1* might be related to the progressive reduction in muscle mass with age [98,99], whereas *smad1* is up-regulated to maximize the use of the available BMP. The other two linked genes, *calcrl* (Calcitonin gene-related peptide type 1 receptor-like) and *ramp1* (Receptor activity-modifying protein 1), encode for two components of G protein-coupled receptors, specifically the calcitonin receptor-like receptor and a receptor activity-modifying protein, respectively. These receptors mediate the effects of peptide hormones, such as calcitonin gene-related peptide (CGRP), which have remarkable roles during adulthood and aging, especially in the neuromuscular system [100,101]. Indeed, in skeletal muscle, CGRP has been reported to potentiate muscle contraction [102] and raise the rate of blood flow locally following muscle contraction [103,104,105]. In this study, the expression of *calcrl* is down-regulated and that of *ramp1*, up-regulated with age. Thus, it may be proposed that increased transcription of *ramp1* might be an effective strategy for increasing the effectiveness of CGRP-induced effects [106], so its expression may be increased with age to compensate the reduction in *calcrl* transcription. Another down-regulated gene was *thrb* (thyroid hormone receptor beta), which encodes for a nuclear hormone receptor for thyroid hormones that participate in skeletal muscle contractile function, myogenesis and muscle regeneration [107]. Thus, its down-regulation may be related to the progressive decline in muscle activity with age [98,99,108].

In our experimental model, another candidate marker that was down-regulated with advancing age is *col5a1*, (collagen alpha-1(V) chain). Collagen is the major structural protein in the extracellular matrix (ECM) of the skeletal muscle and it is essential for the mechanical support of tissue, although the gene expression of ECM components, including collagenic genes, decreases with age [63,109]. Likewise, the gene *spred2* (sprouty-related, EVH1 domain-containing protein 2) encodes for one of the proteins that control growth factor-induced activation of the mitogen-activated protein kinase (MAPK) cascade [110], which is considered a positive regulator of muscle atrophy [111]. Hence, the lower expression of this gene in older animals, and consequently a lower inhibition of MAPK, could be the cause of increased muscle atrophy with age. Finally, *atp1a2* (sodium/potassium-transporting ATPase subunit alpha-2) is one of the genes encoding for different Na^+^, K^+^ ATPase (NKA) α-isoforms and is mainly expressed in muscle, either in mammals [112,113] or fish [114,115,116]. As a euryhaline teleost, gilthead sea bream may exhibit high NKA activity in seawater [117,118,119,120], and the lower expression of this gene might be linked with the reduction in ATPase activity in muscle with age [121].

**Figure 6:**
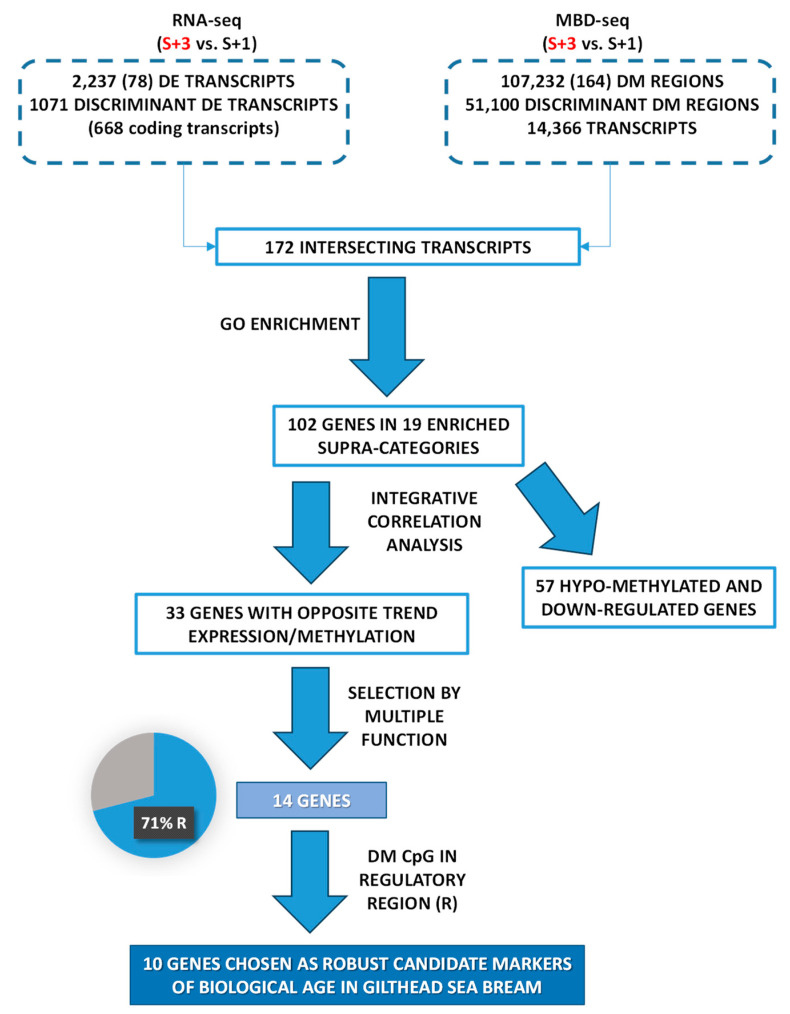
Chart that represents the filters and criteria that were applied for matching wide transcriptomics (RNA-seq) and wide genomic DNA methylation (MBD-seq) approaches in order to record candidate markers of biological age in gilthead sea bream.

In summary, as depicted in Figure 6, an integrative approach of transcriptomics and DNA methylation data from one- and three-year-old fish applied over successive selection steps enabled generating a candidate list of muscle markers of biological age in farmed gilthead sea bream. Previous attempts of age-related markers in fisheries and conservation biology studies have been focused on chronological age markers, but in farmed animals attention is now moved towards indicators of the cumulative burden of environmental experiences, which may accelerate or decelerate the age progression along the production cycle. In comparison to common biomarkers of age, such as telomere length, DNA methylation marks very often have a better predictive ability [122], which would be reinforced herein by the integration of two omics layer (transcriptomics and epigenetics), followed by the use of successive filters to disclose age-related markers highly influenced by cumulative life insults. The result is a reduced list of 10 potential biological age markers that require validation in independent studies. Afterwards, the generated knowledge (i.e., the gene expression data from these biomarkers) might be applied in samples from a wide range of age groups (from very young to adult) through an appropriate estimation algorithm (e.g., multiple linear regression) [123,124] to compute individual’s biological age at the field scale as part of an improved welfare assessment system for auditing farmed fish welfare.

## 4. Materials and Methods

### 4.1. Ethics Statement

All procedures of animal rearing and sampling were conducted according to the current IATS-CSIC Review Board, European animal directives (2010/63/EU), and Spanish laws (Royal Decree RD53/2013) for the protection of animals used in scientific experiments.

### 4.2. Experimental Setup

One- (S + 1) and three- (S + 3) year-old gilthead sea bream (GSB) of Atlantic origin were reared at the indoor experimental facilities of the Institute of Aquaculture Torre de la Sal (IATS-CSIC) in 3000 L tanks under natural photoperiod and temperature conditions at the IATS-CSIC latitude (40°5 N; 0°10 E). Water temperature was between 23 and 28 °C in the summer when sampling was conducted, the water oxygen concentration was maintained over 75% saturation, and unionized ammonia remained below toxic levels (<0.02 mg/L). Fish received a standard commercial diet (EFICO YM 568; BioMar, Dueñas, Spain) once a day until visual satiety (5 or 6 times per week depending on fish size). Seven fish per age class (one year old, S + 1, 100–115 g body weight; three years old, S + 3, 1 kg body weight) were anesthetized with 3-aminobenzoic acid ethyl ester (MS-222, 100 μg/mL), and white skeletal muscle (WSM) was rapidly excised, frozen in liquid nitrogen and stored at −80 °C until RNA and DNA extraction.

### 4.3. DNA/RNA Extraction

White skeletal muscle DNA was obtained using the Quick-DNA™ Mini-prep Plus Kit (Zymo Research, Irvine, CA, USA) based on the manufacturer’s instructions. Assessment of the quantity and quality of DNA was carried out by a NanoDrop 2000cSpectrophotometer (Thermo Fisher Scientific, Waltham, MA, USA), and DNA integrity was evaluated in a 1% agarose gel. Total RNA (70–100 μg) from WSM was extracted with the MagMAXTM-96 Total RNA Isolation Kit (Applied Biosystems, Foster City, CA, USA). A Nanodrop 2000c Spectrophotometer (Thermo Fisher Scientific, Waltham, MA, USA) was employed to determine the RNA concentration and purity. The quality and integrity of this isolated RNA were established on an Agilent Bioanalyzer 2100 total RNA Nano series II chip (Agilent, Amstelveen, The Netherlands), yielding adequate RNA integrity numbers (RINs; 8–10). Samples were stored at −80 °C until DNA and RNA sequencing.

### 4.4. DNA/RNA Illumina Sequencing

For the methyl-binding domain sequencing (MBD-seq) analysis, 300 ng of DNA from S + 1 and S + 3 samples was fragmented to 200–550 pb using the methylation-insensitive restriction enzyme MseI (New England Biolabs, Ipswich, MA, USA), which binds genomic T↓TAA sites, typically located outside of CGIs. The enzyme action and inactivation temperatures, together with its actuation times and concentration were fixed according to the manufacturer’s instructions. Products, obtained using AMPure beads were checked and quantified with Picogreen (Invitrogen, Carlsbad, CA, USA). Fragments of DNA were then submitted to methylation enrichment through the MethylCollectorTM Ultra kit (Active Motif, Carlsbad, CA, USA), based on the instructions. Briefly, methylated DNA was collected from 75 ng of fragmented DNA via attaching to the methyl-CpG binding domain of the MBD2 protein. Libraries for Illumina MBD-seq were obtained from 15 ng of methylated DNA fragments employing the NEBNext^®^ Ultra™ II DNA Library Prep Kit (Illumina Inc., San Diego, CA, USA) according to the manufacturer’s indications. All libraries were sequenced on an Illumina NextSeq 500 HO sequencer as a 1 × 75 nucleotides single-end (SE) read format, following the manufacturer’s protocol. Raw sequenced data were deposited in the Sequence Read Archive (SRA) of the National Center for Biotechnology Information (NCBI) under the Bioproject accession number PRJNA1096613 (BioSample accession numbers: SAMN40757284-297).

Illumina RNA-seq libraries were acquired from 500 ng total RNA using the Illumina TruSeq™ Stranded mRNA LT Sample Prep Kit (Illumina Inc., San Diego, CA, USA) according to the manufacturer’s indications. All RNA-seq libraries were sequenced through an Illumina NextSeq 500 HO sequencer as 1 × 75 nucleotides SE read format according to the manufacturer’s instructions. Raw sequenced data were deposited in the Sequence Read Archive (SRA) of the National Center for Biotechnology Information (NCBI) under the Bioproject accession number PRJNA1096613 (BioSample accession numbers: SAMN40757298-311).

### 4.5. Bioinformatic Analyses

After sequencing, evaluation of the quality of the RNA-seq and MBD-seq resulting raw reads was carried out with FASTQC (https://www.bioinformatics.babraham.ac.uk/projects/fastqc/; accessed on: 21 November 2022). RNA-seq libraries were filtered with Prinseq [125] eliminating those with quality < 18, length < 60 bp, and > 5% of Ns in the sequence. Cleaned reads were mapped against gilthead sea bream reference genome [126] (available at https://seabreamdb.nutrigroup-iats.org/; accessed on: 21 November 2022), using TopHat v2.1.1 [127]. A representative transcriptome per sample was created using Cufflinks v0.17.3, being data quality-checked with CummeRbund v2.7.2 [128].

In the case of MBD-seq data, pre-processing was conducted with Prinseq [125], eliminating those with quality < 20, length < 60 bp, and >5% of Ns in the sequence. Before mapping, the repeat regions of the gilthead sea bream reference were masked using the BSgenome 1.72.0 R package. Then, high-quality reads were aligned to this masked genome through the Bowtie2 v2.3.4.3 software [129]. After MBD-seq mapping, the methylated reads were located in their corresponding genomic region employing five training sets created ad hoc for this analysis. The exon and intron training sets contained the coordinates of these elements in the genome. The promoter region of a gene was considered 5000 bp before its start codon. Finally, two training sets were established for CGI, relying on if these islands were located in promoters or intergenic regions. Predictions of CGI were performed with the NewCpGReport tool from the EMBOSS suite [130]. Search parameters for CGI were: length ≥ 200, C + G content ≥ 50%, ratio of observed/expected CpGs ≥ 0.60 and window size = 100.

### 4.6. Statistical Analysis and Data Filtering

**Figure 7:**
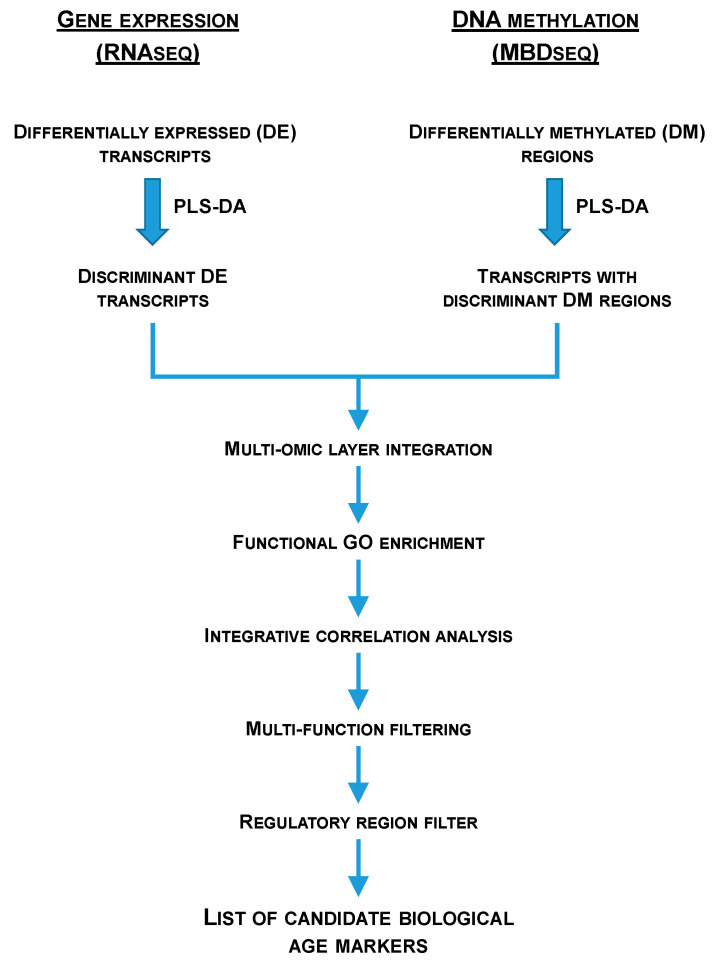
Flow chart representing the multiomic layer integration and the different bioinformatic analysis and filters applied after wide transcriptomics (RNA-seq) and wide genomic DNA methylation (MBD-seq) approaches in order to obtain a list of candidate markers of biological age in gilthead sea bream.

RNA-seq data were first normalized in FPKMs using Cufflinks [127]. Then, DE transcripts in RNA-seq data were recovered using the Wald test implemented in DESeq2 at two significance thresholds (*p* < 0.05 and FDR < 0.05) [131]. MBD-seq data were normalized at reads per kilobase per million (RPKMS) and DM regions in MBD-seq data were retrieved using the MEDIPS R package release 3.19 (*p* < 0.05) [132] over 25 bp size regions, containing at least one dinucleotide CG. Several partial least-squares discriminant analyses (PLS-DA), using EZinfo v3.0 (Umetrics, Umeå, Sweden), were performed to study the separation of the experimental groups. Significant (*p* < 0.05) DE transcripts and DM regions were introduced in the analyses. The fitness and predictability of these models were validated by a 500 random permutation test (pR2Y < 0.05; pQ2 < 0.05) using the ropls R package [133]. The discriminant ability of each marker was ranked after the creation of the models based on its Variable Importance in the Projection (VIP), and useful markers were found under a VIP ≥ 1 [134]. Discriminant DE transcripts were matched with those transcripts containing discriminant DM regions (multiomic layer integration) and different bioinformatic analysis and filters (i.e., functional GO enrichment, integrative correlation analysis, multifunction filtering, regulatory region—either promoter, first intron or first exon—filter) were applied over the obtained overlapping transcripts (Figure 7) in order to obtain the list of candidate biological age markers. Over-representation tests of GO-BP terms were implemented in the ShinyGO 0.77 web application [135] and statistical significance was accepted at FDR < 0.05. GO-BP levels and supra-categories were obtained using GOATools [136]. Pearson correlation coefficients between the expression and methylation Log2FC were conducted using the SigmaPlot v14.5 (Systat Software Inc., San Jose, CA, USA) software. Representations of the gene structure were obtained using the genemodel R package (https://github.com/greymonroe/genemodel; accessed on: 21 November 2022) and the IBS software v1.0.3 [137].

## Figures and Tables

**Table 1:** Functional processes (GO-BP ancestors) associated to the selected genes with opposite effect of DNA methylation and expression (33), ordered by the number of selected linked genes (*n*). The name of these genes and the corresponding age-related changes in DNA methylation and expression are also presented (genes in green, hyper-methylated and down-regulated; gene in red, hypo-methylated and up-regulated). Met., metabolic; Cel., cellular; Reg., regulation; Multicel., multicellular.

Functional Process	*n*	Genes
System development	17	*abi3bp*, *arhgap24*, *bmp1*, *calcrl*, *col5a1*, *kcp*, *mical2*, *myo18b*, *nrp2*, *spred2*, *thrb*, *wfikkn2*, *parp3* , *psmd2*, *ramp1*, *sirt1*, *smad1*
Anatomical structure development	12	*arhgap24* , *calcrl*, *col5a1*, *kidins220*, *mical2*, *myo18b*, *nrp2*, *thrb*, * phf6* , *ramp1*, *sirt1*, *smad1*
Anatomical structure morphogenesis	12	*arhgap24* , *bmp1*, *calcrl*, *col5a1*, *mical2*, *myo18b*, *nrp2*, *thrb*, *psmd2* , *ramp1*, *sirt1*, *smad1*
Reg. multicel. organismal process	11	*abi3bp*, *atp1a2*, *bmp1*, *calcrl*, *sema6d*, *spred2*, *zbtb20*, * parp3* , *scn3b*, *sirt1*, *smad1*
Cel. component organization	10	*abi3bp*, *bmp1*, *col5a1*, *col6a3*, *colgalt1*, *kirrel1*, *mical2*, *nrp2*, *olfml2a*, *myoz2*
Response to endogenous stimulus	10	*atp1a2*, *kcp*, *kidins220*, *nrp2*, *spred2*, *thrb*, *wfikkn2*, *ramp1* , *sirt1*, *smad1*
Animal organ development	9	*abi3bp*, *bmp1*, *calcrl*, *col5a1*, *mical2*, *nrp2*, *spred2*, *thrb*, *smad1*
Reg. cel. process	9	*abi3bp*, *calcrl*, *col5a1*, *colgalt*, *ksr1*, *nrp2*, *ramp1*, *sirt1*, *smad1*
Anatomical structure formation	8	*arhgap24* , *calcrl*, *col5a1*, *nrp2*, * myoz2* , *ramp1*, *sirt1*, *smad1*
Reg. developmental process	8	*abi3bp*, *bmp1*, *col5a1*, *sema6d*, *spred2*, *psmd2* , *sirt1*, *smad1*
Reg. localization	4	*atp1a2*, *fgf14*, *hecw2*, *scn3b*
Tissue development	4	*bmp1* , *col5a1*, *sirt1* , *smad1*
Transport	4	*atp1a2*, *fgf14*, *hecw2*, *scn3b*
Response to chemical	3	*calcrl*, *zbtb20*, *sirt1*
N compound met. process	2	*psmd2* , *srm*
Ossification	2	*bmp1* , *smad1*
Rhythmic process	2	*tef* , *sirt1*
Cell differentiation	1	*col5a1*

**Table 2:** Selected genes with opposite effect of DNA methylation and expression (33) ordered by the number of functional processes (GO-BP ancestors; n) they are involved (genes in green, hyper-methylated and down-regulated; gene in red, hypo-methylated and up-regulated; genes in bold, containing methylated CpG in a regulating area). The names of the corresponding functional processes are also presented. Met., metabolic; Cel., cellular; Reg., regulation; Multicel., multicellular.

Genes	n	Functional Processes
***sirt1***	11	Anatomical structure development, Anatomical structure formation, Anatomical structure morphogenesis, Reg. cel. process, Reg. developmental process, Reg. multicel. organismal process, Response to chemical, Response to endogenous stimulus, Rhythmic process, System development, Tissue development
***smad1***	11	Anatomical structure development, Anatomical structure formation, Anatomical structure morphogenesis, Animal organ development, Ossification, Reg. cel. process, Reg. developmental process, Reg. multicel. organismal process, Response to endogenous stimulus, System development, Tissue development
***col5a1***	10	Anatomical structure development, Anatomical structure formation, Anatomical structure morphogenesis, Animal organ development, Cell differentiation, Cel. component organization, Reg. cel. process, Reg. developmental process, System development, Tissue development
***calcrl***	8	Anatomical structure development, Anatomical structure formation, Anatomical structure morphogenesis, Animal organ development, Reg. cel. process, Reg. multicel. organismal process, Response to chemical, System development
*nrp2*	8	Anatomical structure development, Anatomical structure formation, Anatomical structure morphogenesis, Animal organ development, Cel. component organization, Reg. cel. process, Response to endogenous stimulus, System development
***bmp1***	8	Anatomical structure morphogenesis, Animal organ development, Cel. Component organization, Ossification, Reg. developmental process, Reg. multicel. Organismal process, System development, Tissue development
***ramp1***	6	Anatomical structure development, Anatomical structure formation, Anatomical structure morphogenesis, Reg. cel. process, Response to endogenous stimulus, System development
*abi3bp*	6	Animal organ development, Cel. component organization, Reg. cel. process, Reg. developmental process, Reg. multicel. organismal process, System development
*mical2*	5	Anatomical structure development, Anatomical structure morphogenesis, Animal organ development, Cel. component organization, System development
***thrb***	5	Anatomical structure development, Anatomical structure morphogenesis, Animal organ development, Response to endogenous stimulus, System development
***spred2***	5	Animal organ development, Reg. developmental process, Reg. multicel. organismal process, Response to endogenous stimulus, System development
*arhgap24*	4	Anatomical structure development, Anatomical structure formation, Anatomical structure morphogenesis, System development
***psmd2***	4	Anatomical structure morphogenesis, N compound met. process, Reg. developmental process, System development
***atp1a2***	4	Reg. localization, Reg. multicel. organismal process, Response to endogenous stimulus, Transport

## Data Availability

Most data generated or analyzed during this study are included in this article and its Supplementary Information Files. Raw sequenced data were deposited in the Sequence Read Archive (SRA) of the National Center for Biotechnology Information (NCBI) under the Bioproject accession numbers PRJNA1096613 (BioSample accession numbers: SAMN40757284-297) and PRJNA1096613 (BioSample accession numbers: SAMN40757298-311).

## Supplementary Materials

The following supporting information can be downloaded at: https://www.mdpi.com/article/10.3390/ijms25189836/s1.

## References

[1] Ahmad A., Sheikh Abdullah S.R., Hasan H.A., Othman A.R., Ismail N. (2021). Izzati Aquaculture Industry: Supply and Demand, Best Practices, Effluent and Its Current Issues and Treatment Technology. J. Environ. Manag..

[2] Naylor R.L., Hardy R.W., Buschmann A.H., Bush S.R., Cao L., Klinger D.H., Little D.C., Lubchenco J., Shumway S.E., Troell M. (2021). A 20-Year Retrospective Review of Global Aquaculture. Nature.

[3] North B.P., Turnbull J.F., Ellis T., Porter M.J., Migaud H., Bron J., Bromage N.R. (2006). The Impact of Stocking Density on the Welfare of Rainbow Trout (*Oncorhynchus mykiss*). Aquaculture.

[4] Liu B., Liu Y., Sun G. (2017). Effects of Stocking Density on Growth Performance and Welfare-Related Physiological Parameters of Atlantic Salmon Salmo Salar, L. in Recirculating Aquaculture System. Aquac. Res..

[5] Wu F., Wen H., Tian J., Jiang M., Liu W., Yang C., Yu L., Lu X. (2018). Effect of Stocking Density on Growth Performance, Serum Biochemical Parameters, and Muscle Texture Properties of Genetically Improved Farm Tilapia, *Oreochromis niloticus*. Aquac. Int..

[6] Jennings S., Stentiford G.D., Leocadio A.M., Jeffery K.R., Metcalfe J.D., Katsiadaki I., Auchterlonie N.A., Mangi S.C., Pinnegar J.K., Ellis T. (2016). Aquatic Food Security: Insights into Challenges and Solutions from an Analysis of Interactions between Fisheries, Aquaculture, Food Safety, Human Health, Fish and Human Welfare, Economy and Environment. Fish. Fish..

[7] Raposo de Magalhães C.S.F., Cerqueira M.A.C., Schrama D., Moreira M.J.V., Boonanuntanasarn S., Rodrigues P.M.L. (2020). A Proteomics and Other Omics Approach in the Context of Farmed Fish Welfare and Biomarker Discovery. Rev. Aquac..

[8] Stien L.H., Bracke M.B.M., Folkedal O., Nilsson J., Oppedal F., Torgersen T., Kittilsen S., Midtlyng P.J., Vindas M.A., Øverli Ø. (2013). Salmon Welfare Index Model (SWIM 1.0): A Semantic Model for Overall Welfare Assessment of Caged Atlantic Salmon: Review of the Selected Welfare Indicators and Model Presentation. Rev. Aquac..

[9] Noble C., Gismervik K., Iversen M.H., Kolarevic J., Nilsson J., Stien L.H., Turnbull J.F. (2018). Welfare Indicators for Farmed Atlantic Salmon: Tools for Assessing Fish Welfare.

[10] Noble C., Gismervik K., Iversen M.H., Kolarevic J., Nilsson J., Stien L.H., Turnbull J.F. (2020). Welfare Indicators for Farmed Rainbow Trout: Tools for Assessing Fish Welfare.

[11] Sadoul B., Geffroy B. (2019). Measuring Cortisol, the Major Stress Hormone in Fishes. J. Fish. Biol..

[12] Winberg S., Höglund E., Øverli Ø. (2016). Variation in the Neuroendocrine Stress Response. Fish Physiology.

[13] Madaro A., Nilsson J., Whatmore P., Roh H.J., Grove S., Stien L.H., Olsen R.E. (2023). Acute Stress Response on Atlantic Salmon: A Time-Course Study of the Effects on Plasma Metabolites, Mucus Cortisol Levels, and Head Kidney Transcriptome Profile. Fish. Physiol. Biochem..

[14] Matley J.K., Johansen L.K., Klinard N.V., Eanes S.T., Jobsis P.D. (2021). Habitat Selection and 3D Space Use Partitioning of Resident Juvenile Hawksbill Sea Turtles in a Small Caribbean Bay. Mar. Biol..

[15] Georgopoulou D.G., Vouidaskis C., Papandroulakis N. (2024). Swimming Behavior as a Potential Metric to Detect Satiation Levels of European Seabass in Marine Cages. Front. Mar. Sci..

[16] Calduch-Giner J., Holhorea P.G., Ferrer M.A., Naya-Català F., Rosell-Moll E., Vega García C., Prunet P., Espmark A.M., Leguen I., Kolarevic J. (2022). Revising the Impact and Prospects of Activity and Ventilation Rate Bio-Loggers for Tracking Welfare and Fish-Environment Interactions in Salmonids and Mediterranean Farmed Fish. Front. Mar. Sci..

[17] Bozzi D., Rasmussen J.A., Carøe C., Sveier H., Nordøy K., Gilbert M.T.P., Limborg M.T. (2021). Salmon Gut Microbiota Correlates with Disease Infection Status: Potential for Monitoring Health in Farmed Animals. Anim. Microbiome.

[18] Domingo-Bretón R., Cools S., Belenguer A., Calduch-Giner J.A., Croes E., Holhorea P.G., Naya-Català F., Boon H., Pérez-Sánchez J. Gilthead Sea Bream Microbiota Shifts Associated with Thermal Stress and Dietary Intervention during a Record Heat Summer. Proceedings of the Aquaculture Europe 2023.

[19] Steiner K., Laroche O., Walker S.P., Symonds J.E. (2022). Effects of Water Temperature on the Gut Microbiome and Physiology of Chinook Salmon (*Oncorhynchus tshawytscha*) Reared in a Freshwater Recirculating System. Aquaculture.

[20] Montero D., Rimoldi S., Torrecillas S., Rapp J., Moroni F., Herrera A., Gómez M., Fernández-Montero Á., Terova G. (2022). Impact of Polypropylene Microplastics and Chemical Pollutants on European Sea Bass (*Dicentrarchus labrax*) Gut Microbiota and Health. Sci. Total Environ..

[21] Bao R., Cheng Z., Peng L., Mehmood T., Gao L., Zhuo S., Wang L., Su Y. (2023). Effects of Biodegradable and Conventional Microplastics on the Intestine, Intestinal Community Composition, and Metabolic Levels in Tilapia (*Oreochromis mossambicus*). Aquat. Toxicol..

[22] Toxqui-Rodríguez S., Naya-Català F., Sitjà-Bobadilla A., Piazzon M.C., Pérez-Sánchez J. (2023). Fish Microbiomics: Strengths and Limitations of MinION Sequencing of Gilthead Sea Bream (*Sparus aurata*) Intestinal Microbiota. Aquaculture.

[23] Older C.E., Yamamoto F.Y., Griffin M.J., Ware C., Heckman T.I., Soto E., Bosworth B.G., Waldbieser G.C. (2024). Comparison of High-Throughput Sequencing Methods for Bacterial Microbiota Profiling in Catfish Aquaculture. N. Am. J. Aquac..

[24] Sun F., Wang Y., Wang C., Zhang L., Tu K., Zheng Z. (2019). Insights into the Intestinal Microbiota of Several Aquatic Organisms and Association with the Surrounding Environment. Aquaculture.

[25] Naya-Català F., Piazzon M.C., Torrecillas S., Toxqui-Rodríguez S., Calduch-Giner J.A., Fontanillas R., Sitjà-Bobadilla A., Montero D., Pérez-Sánchez J. (2022). Genetics and Nutrition Drive the Gut Microbiota Succession and Host-Transcriptome Interactions through the Gilthead Sea Bream (*Sparus aurata*) Production Cycle. Biology.

[26] Horvath S. (2013). DNA Methylation Age of Human Tissues and Cell Types. Genome Biol..

[27] De Paoli-Iseppi R., Deagle B.E., Polanowski A.M., McMahon C.R., Dickinson J.L., Hindell M.A., Jarman S.N. (2019). Age Estimation in a Long-Lived Seabird (Ardenna Tenuirostris) Using DNA Methylation-Based Biomarkers. Mol. Ecol. Resour..

[28] Piferrer F., Anastasiadi D. (2023). Age Estimation in Fishes Using Epigenetic Clocks: Applications to Fisheries Management and Conservation Biology. Front. Mar. Sci..

[29] O’Dea R.E., Noble D.W.A., Johnson S.L., Hesselson D., Nakagawa S. (2016). The Role of Non-Genetic Inheritance in Evolutionary Rescue: Epigenetic Buffering, Heritable Bet Hedging and Epigenetic Traps. Environ. Epigenet.

[30] Parrott B.B., Bertucci E.M. (2019). Epigenetic Aging Clocks in Ecology and Evolution. Trends Ecol. Evol..

[31] Bertucci E.M., Wason M.W., Rhodes O.E., Parrott B.B. (2021). Exposure to ionizing radiation disrupts normal epigenetic aging in Japanese medaka. Aging.

[32] Bell C.G., Lowe R., Adams P.D., Baccarelli A.A., Beck S., Bell J.T., Christensen B.C., Gladyshev V.N., Heijmans B.T., Horvath S. (2019). DNA Methylation Aging Clocks: Challenges and Recommendations. Genome Biol..

[33] Gensous N., Sala C., Pirazzini C., Ravaioli F., Milazzo M., Kwiatkowska K.M., Marasco E., De Fanti S., Giuliani C., Pellegrini C. (2022). A Targeted Epigenetic Clock for the Prediction of Biological Age. Cells.

[34] Rutledge J., Oh H., Wyss-Coray T. (2022). Measuring Biological Age Using Omics Data. Nat. Rev. Genet..

[35] Duan R., Fu Q., Sun Y., Li Q. (2022). Epigenetic Clock: A Promising Biomarker and Practical Tool in Aging. Ageing Res. Rev..

[36] Tangili M., Slettenhaar A.J., Sudyka J., Dugdale H.L., Pen I., Palsbøll P.J., Verhulst S. (2023). DNA Methylation Markers of Age(Ing) in Non-Model Animals. Mol. Ecol..

[37] Bateson M., Poirier C. (2019). Can Biomarkers of Biological Age Be Used to Assess Cumulative Lifetime Experience?. Anim. Welf..

[38] Keeling L.J., Winckler C., Hintze S., Forkman B. (2021). Towards a Positive Welfare Protocol for Cattle: A Critical Review of Indicators and Suggestion of How We Might Proceed. Front. Anim. Sci..

[39] da Silva A.N., Araujo M.S., Pértille F., Zanella A.J. (2022). How Epigenetics Can Enhance Pig Welfare?. Animals.

[40] Colditz I.G., Smith E.G., Ingham A.B., Dominik S. (2023). Indicators of Functional Integrity in Production Animals. Anim. Prod. Sci..

[41] Simpson D.J., Chandra T. (2021). Epigenetic Age Prediction. Aging Cell.

[42] Jung S., Arcos Hodar J., del Sol A. (2023). Measuring Biological Age Using a Functionally Interpretable Multi-Tissue RNA Clock. Aging Cell.

[43] Naya-Català F., Belenguer A., Montero D., Torrecillas S., Soriano B., Calduch-Giner J., Llorens C., Fontanillas R., Sarih S., Zamorano M.J. (2023). Broodstock Nutritional Programming Differentially Affects the Hepatic Transcriptome and Genome-Wide DNA Methylome of Farmed Gilthead Sea Bream (*Sparus aurata*) Depending on Genetic Background. BMC Genom..

[44] Valdivieso A., Anastasiadi D., Ribas L., Piferrer F. (2023). Development of Epigenetic Biomarkers for the Identification of Sex and Thermal Stress in Fish Using DNA Methylation Analysis and Machine Learning Procedures. Mol. Ecol. Resour..

[45] Beemelmanns A., Ribas L., Anastasiadi D., Moraleda-Prados J., Zanuzzo F.S., Rise M.L., Gamperl A.K. (2021). DNA Methylation Dynamics in Atlantic Salmon (*Salmo salar*) Challenged With High Temperature and Moderate Hypoxia. Front. Mar. Sci..

[46] Simó-Mirabet P., Perera E., Calduch-Giner J.A., Pérez-Sánchez J. (2020). Local DNA Methylation Helps to Regulate Muscle Sirtuin 1 Gene Expression across Seasons and Advancing Age in Gilthead Sea Bream (*Sparus aurata*). Front. Zool..

[47] Li Y., Huang J., Liu Z., Zhou Y., Xia B., Wang Y., Kang Y., Wang J. (2017). Transcriptome Analysis Provides Insights into Hepatic Responses to Moderate Heat Stress in the Rainbow Trout (*Oncorhynchus mykiss*). Gene.

[48] Beemelmanns A., Zanuzzo F.S., Xue X., Sandrelli R.M., Rise M.L., Gamperl A.K. (2021). The Transcriptomic Responses of Atlantic Salmon (*Salmo Salar*) to High Temperature Stress Alone, and in Combination with Moderate Hypoxia. BMC Genom..

[49] Burgerhout E., Mommens M., Johnsen H., Aunsmo A., Santi N., Andersen O. (2017). Genetic Background and Embryonic Temperature Affect DNA Methylation and Expression of Myogenin and Muscle Development in Atlantic Salmon (Salmo Salar). PLoS ONE.

[50] Veron V., Marandel L., Liu J., Vélez E.J., Lepais O., Panserat S., Skiba S., Seiliez I. (2018). DNA Methylation of the Promoter Region of *Bnip3* and *Bnip3l* Genes Induced by Metabolic Programming 06 Biological Sciences 0604 Genetics. BMC Genom..

[51] Zheng J.L., Guo S.N., Yuan S.S., Xia H., Zhu Q.L., Lv Z.M. (2017). Preheating Mitigates Cadmium Toxicity in Zebrafish Livers: Evidence from Promoter Demethylation, Gene Transcription to Biochemical Levels. Aquat. Toxicol..

[52] Stien L.H., Bracke M., Noble C., Kristiansen T.S., Kristiansen T.S., Ferno A., Pavlidis M.A., van de Vis H. (2020). Assessing Fish Welfare in Aquaculture. The Welfare of the Fish.

[53] van de Vis H., Kolarevic J., Stien L.H., Kristiansen T.S., Gerritzen M., van de Braak K., Abbink W., Sæther B.-S., Noble C., Kristiansen T.S., Ferno A., Pavlidis M.A., van de Vis H. (2020). Welfare of Farmed Fish in Different Production Systems and Operations. The Welfare of the Fish.

[54] Piferrer F., Blázquez M., Navarro L., González A. (2005). Genetic, Endocrine, and Environmental Components of Sex Determination and Differentiation in the European Sea Bass (*Dicentrarchus labrax* L.). Gen. Comp. Endocrinol..

[55] Navarro-Martín L., Blázquez M., Viñas J., Joly S., Piferrer F. (2009). Balancing the Effects of Rearing at Low Temperature during Early Development on Sex Ratios, Growth and Maturation in the European Sea Bass (*Dicentrarchus labrax*). Limitations and Opportunities for the Production of Highly Female-Biased Stocks. Aquaculture.

[56] Simó-Mirabet P., Felip A., Estensoro I., Martos-Sitcha J.A., de las Heras V., Calduch-Giner J., Puyalto M., Karalazos V., Sitjà-Bobadilla A., Pérez-Sánchez J. (2018). Impact of Low Fish Meal and Fish Oil Diets on the Performance, Sex Steroid Profile and Male-Female Sex Reversal of Gilthead Sea Bream (*Sparus aurata*) over a Three-Year Production Cycle. Aquaculture.

[57] Holhorea P.G., Felip A., Calduch-Giner J.A., Afonso J.M., Pérez-Sánchez J. (2023). Use of Male-to-Female Sex Reversal as a Welfare Scoring System in the Protandrous Farmed Gilthead Sea Bream (*Sparus aurata*). Front. Vet. Sci..

[58] Frenk S., Houseley J. (2018). Gene Expression Hallmarks of Cellular Ageing. Biogerontology.

[59] López-Otín C., Blasco M.A., Partridge L., Serrano M., Kroemer G. (2023). Hallmarks of Aging: An Expanding Universe. Cell.

[60] Stegeman R., Weake V.M. (2017). Transcriptional Signatures of Aging. J. Mol. Biol..

[61] Shavlakadze T., Morris M., Fang J., Wang S.X., Zhu J., Zhou W., Tse H.W., Mondragon-Gonzalez R., Roma G., Glass D.J. (2019). Age-Related Gene Expression Signature in Rats Demonstrate Early, Late, and Linear Transcriptional Changes from Multiple Tissues. Cell Rep..

[62] Hernando-Herraez I., Evano B., Stubbs T., Commere P.H., Jan Bonder M., Clark S., Andrews S., Tajbakhsh S., Reik W. (2019). Ageing Affects DNA Methylation Drift and Transcriptional Cell-to-Cell Variability in Mouse Muscle Stem Cells. Nat. Commun..

[63] Kijima Y., Wantong W., Igarashi Y., Yoshitake K., Asakawa S., Suzuki Y., Watabe S., Kinoshita S. (2022). Age-Associated Different Transcriptome Profiling in Zebrafish and Rats: An Insight into the Diversity of Vertebrate Aging. Mar. Biotechnol..

[64] Xu A., Teefy B.B., Lu R.J., Nozownik S., Tyers A.M., Valenzano D.R., Benayoun B.A. (2023). Transcriptomes of Aging Brain, Heart, Muscle, and Spleen from Female and Male African Turquoise Killifish. Sci. Data.

[65] López-Otín C., Blasco M.A., Partridge L., Serrano M., Kroemer G. (2013). The Hallmarks of Aging. Cell.

[66] López-Gil L., Pascual-Ahuir A., Proft M. (2023). Genomic Instability and Epigenetic Changes during Aging. Int. J. Mol. Sci..

[67] Jung M., Pfeifer G.P. (2015). Aging and DNA Methylation. BMC Biol..

[68] Zupkovitz G., Kabiljo J., Kothmayer M., Schlick K., Schöfer C., Lagger S., Pusch O. (2021). Analysis of Methylation Dynamics Reveals a Tissue-Specific, Age-Dependent Decline in 5-Methylcytosine Within the Genome of the Vertebrate Aging Model *Nothobranchius furzeri*. Front. Mol. Biosci..

[69] Heyn H., Li N., Ferreira H.J., Moran S., Pisano D.G., Gomez A., Diez J., Sanchez-Mut J.V., Setien F., Carmona F.J. (2012). Distinct DNA Methylomes of Newborns and Centenarians. Proc. Natl. Acad. Sci. USA.

[70] Johnson A.A., Akman K., Calimport S.R.G., Wuttke D., Stolzing A., De Magalhães J.P. (2012). The Role of DNA Methylation in Aging, Rejuvenation, and Age-Related Disease. Rejuvenation Res..

[71] Shimoda N., Izawa T., Yoshizawa A., Yokoi H., Kikuchi Y., Hashimoto N. (2014). Decrease in Cytosine Methylation at CpG Island Shores and Increase in DNA Fragmentation during Zebrafish Aging. Age.

[72] Anastasiadi D., Piferrer F. (2020). A Clockwork Fish: Age Prediction Using DNA Methylation-Based Biomarkers in the European Seabass. Mol. Ecol. Resour..

[73] Newell-Price J., Adrian J.L.C., King P. (2000). DNA Methylation and Silencing of Gene Expression. Trends Endocrinol. Metab..

[74] Biermann K., Steger K. (2007). Epigenetics in Male Germ Cells. J. Androl..

[75] Jones P.A. (2012). Functions of DNA Methylation: Islands, Start Sites, Gene Bodies and Beyond. Nat. Rev. Genet..

[76] Moore L.D., Le T., Fan G. (2013). DNA Methylation and Its Basic Function. Neuropsychopharmacology.

[77] Brenet F., Moh M., Funk P., Feierstein E., Viale A.J., Socci N.D., Scandura J.M. (2011). DNA Methylation of the First Exon Is Tightly Linked to Transcriptional Silencing. PLoS ONE.

[78] Anastasiadi D., Esteve-Codina A., Piferrer F. (2018). Consistent Inverse Correlation between DNA Methylation of the First Intron and Gene Expression across Tissues and Species. Epigenet Chromatin.

[79] Hon G.C., Hawkins R.D., Caballero O.L., Lo C., Lister R., Pelizzola M., Valsesia A., Ye Z., Kuan S., Edsall L.E. (2012). Global DNA Hypomethylation Coupled to Repressive Chromatin Domain Formation and Gene Silencing in Breast Cancer. Genome Res..

[80] Yang X., Han H., DeCarvalho D.D., Lay F.D., Jones P.A., Liang G. (2014). Gene Body Methylation Can Alter Gene Expression and Is a Therapeutic Target in Cancer. Cancer Cell.

[81] Kovalchuk I., Kovalchuk I., Kovalchuk O. (2021). Role of DNA Methylation in Genome Stability. Genome Stability: From Virus to Human Application.

[82] Vikeså V., Nankervis L., Hevrøy E.M. (2017). Appetite, Metabolism and Growth Regulation in Atlantic Salmon (*Salmo salar* L.) Exposed to Hypoxia at Elevated Seawater Temperature. Aquac. Res..

[83] Naya-Català F., Martos-Sitcha J.A., de las Heras V., Simó-Mirabet P., Calduch-Giner J., Pérez-Sánchez J. (2021). Targeting the Mild-Hypoxia Driving Force for Metabolic and Muscle Transcriptional Reprogramming of Gilthead Sea Bream (*Sparus aurata*) Juveniles. Biology.

[84] Lu A.T., Fei Z., Haghani A., Robeck T.R., Zoller J.A., Li C.Z., Lowe R., Yan Q., Zhang J., Vu H. (2023). Universal DNA Methylation Age across Mammalian Tissues. Nat. Aging.

[85] Toiber D., Sebastian C., Mostoslavsky R., Yao T.-P., Seto E. (2011). Characterization of Nuclear Sirtuins: Molecular Mechanisms and Physiological Relevance. Histone Deacetylases: The Biology and Clinical Implication.

[86] Bellet M.M., Sassone-Corsi P. (2010). Mammalian Circadian Clock and Metabolism—The Epigenetic Link. J. Cell Sci..

[87] Bosch-Presegué L., Vaquero A. (2015). Sirtuin-Dependent Epigenetic Regulation in the Maintenance of Genome Integrity. FEBS J..

[88] Xiao F.H., Kong Q.P., Perry B., He Y.H. (2016). Progress on the Role of DNA Methylation in Aging and Longevity. Brief. Funct. Genom..

[89] Zhao L., Cao J., Hu K., He X., Yun D., Tong T., Han L. (2020). Sirtuins and Their Biological Relevance in Aging and Age-Related Diseases. Aging Dis..

[90] Kitajima Y., Yoshioka K., Suzuki N. (2020). The Ubiquitin-Proteasome System in Regulation of the Skeletal Muscle Homeostasis and Atrophy: From Basic Science to Disorders. J. Physiol. Sci..

[91] Zeng B.Y., Medhurst A.D., Jackson M., Rose S., Jenner P. (2005). Proteasomal Activity in Brain Differs between Species and Brain Regions and Changes with Age. Mech. Ageing Dev..

[92] Dasuri K., Nguyen A., Zhang L., Fernandez-Kim O.S., Bruce-Keller A.J., Blalock B.A., De Cabo R., Keller J.N. (2009). Comparison of Rat Liver and Brain Proteasomes for Oxidative Stress-Induced Inactivation: Influence of Ageing and Dietary Restriction. Free Radic. Res..

[93] Ferrington D.A., Husom A.D., Thompson L.V. (2005). Altered Proteasome Structure, Function, and Oxidation in Aged Muscle. FASEB J..

[94] Krogan N.J., Lam M.H.Y., Fillingham J., Keogh M.C., Gebbia M., Li J., Datta N., Cagney G., Buratowski S., Emili A. (2004). Proteasome Involvement in the Repair of DNA Double-Strand Breaks. Mol. Cell.

[95] Salah Fararjeh A.F., Al-Khader A., Al-Saleem M., Abu Qauod R. (2021). The Prognostic Significance of Proteasome 26S Subunit, Non-ATPase (PSMD) Genes for Bladder Urothelial Carcinoma Patients. Cancer Inform..

[96] Walsh D.W., Godson C., Brazil D.P., Martin F. (2010). Extracellular BMP-Antagonist Regulation in Development and Disease: Tied up in Knots. Trends Cell Biol..

[97] Sartori R., Schirwis E., Blaauw B., Bortolanza S., Zhao J., Enzo E., Stantzou A., Mouisel E., Toniolo L., Ferry A. (2013). BMP Signaling Controls Muscle Mass. Nat. Genet..

[98] Nilwik R., Snijders T., Leenders M., Groen B.B.L., van Kranenburg J., Verdijk L.B., Van Loon L.J.C. (2013). The Decline in Skeletal Muscle Mass with Aging Is Mainly Attributed to a Reduction in Type II Muscle Fiber Size. Exp. Gerontol..

[99] Ruparelia A.A., Salavaty A., Barlow C.K., Lu Y., Sonntag C., Hersey L., Eramo M.J., Krug J., Reuter H., Schittenhelm R.B. (2024). The African Killifish: A Short-Lived Vertebrate Model to Study the Biology of Sarcopenia and Longevity. Aging Cell.

[100] Hodges-Savola C.A., Fernandez H.L. (1995). A Role for Calcitonin Gene-Related Peptide in the Regulation of Rat Skeletal Muscle G 4 Acetylcholinesterase. Neurosci. Lett..

[101] Fernandez H.L., Chen M., Nadelhaft I., Durr J.A. (2003). Calcitonin Gene-Related Peptides: Their Binding Sites and Receptor Accessory Proteins in Adult Mammalian Skeletal Muscles. Neuroscience.

[102] Lu B., Fu W.-m., Greengard P., Poo M.-m. (1993). Calcitonin gene-related peptide potentiates synaptic responses at developing neuromuscular junction. Lett. Nat..

[103] Arden W.A., Fiscus R.R., Beihn L.D., Derbin M., Oremus R., Gross D.R. (1994). Skeletal Muscle Microcirculatory Response to Rat α-Calcitonin Gene-Related Peptide. Neuropeptides.

[104] Yamada M., Ishikawa T., Fujimori A., Goto K. (1997). Local Neurogenic Regulation of Rat Hindlimb Circulation: Role of Calcitonin Gene-Related Peptide in Vasodilatation after Skeletal Muscle Contraction. Br. J. Pharmacol..

[105] Yamada M., Ishikawa T., Yamanaka A., Fujimori A., Goto K. (1997). Local Neurogenic Regulation of Rat Hindlimb Circulation: CO2-Induced Release of Calcitonin Gene-Related Peptide from Sensory Nerves. Br. J. Pharmacol..

[106] Zhang Z., Dickerson I.M., Russo A.F. (2006). Calcitonin Gene-Related Peptide Receptor Activation by Receptor Activity-Modifying Protein-1 Gene Transfer to Vascular Smooth Muscle Cells. Endocrinology.

[107] Bloise F.F., Cordeiro A., Ortiga-Carvalho T.M. (2018). Role of Thyroid Hormone in Skeletal Muscle Physiology. J. Endocrinol..

[108] Zhou Q., Kerbl-Knapp J., Zhang F., Korbelius M., Kuentzel K.B., Vujić N., Akhmetshina A., Hörl G., Paar M., Steyrer E. (2022). Metabolomic Profiles of Mouse Tissues Reveal an Interplay between Aging and Energy Metabolism. Metabolites.

[109] Chen W.J., Lin I.H., Lee C.W., Chen Y.F. (2021). Aged Skeletal Muscle Retains the Ability to Remodel Extracellular Matrix for Degradation of Collagen Deposition after Muscle Injury. Int. J. Mol. Sci..

[110] Nonami A., Kato R., Taniguchi K., Yoshiga D., Taketomi T., Fukuyama S., Harada M., Sasaki A., Yoshimura A. (2004). Spred-1 Negatively Regulates Interleukin-3-Mediated ERK/Mitogen-Activated Protein (MAP) Kinase Activation in Hematopoietic Cells. J. Biol. Chem..

[111] Yuasa K., Okubo K., Yoda M., Otsu K., Ishii Y., Nakamura M., Itoh Y., Horiuchi K. (2018). Targeted Ablation of P38α MAPK Suppresses Denervation-Induced Muscle Atrophy. Sci. Rep..

[112] Lingrel J.B., Kuntzweiler T. (1994). Na+,K+-ATPase. J. Biol. Chem..

[113] Blanco G., Mercer R.W. (1998). Isozymes of the Na-K-ATPase: Heterogeneity in Structure, Diversity in Function. Am. J. Physiol..

[114] Canfield V.A., Loppin B., Thisse B., Thisse C., Postlethwait J.H., Mohideen M.-A.P.K., Johannes S., Rajarao R., Levenson R. (2002). Na,K-ATPase a and b Subunit Genes Exhibit Unique Expression Patterns during Zebrafish Embryogenesis. Mech. Dev..

[115] Richards J.G., Semple J.W., Bystriansky J.S., Schulte P.M. (2003). Na+/K+-ATPase α-Isoform Switching in Gills of Rainbow Trout (Oncorhynchus Mykiss) during Salinity Transfer. J. Exp. Biol..

[116] Doǧanli C., Kjaer-Sorensen K., Knoeckel C., Beck H.C., Nyengaard J.R., Honoré B., Nissen P., Ribera A., Oxvig C., Lykke-Hartmann K. (2012). The A2Na+/K+-ATPase Is Critical for Skeletal and Heart Muscle Function in Zebrafish. J. Cell Sci..

[117] Jensen M.K., Madsen S.S., Kristiansen R. (1998). Osmoregulation and Salinity Effects on the Expression and Activity of Na+,K+-ATPase in the Gills of European Sea Bass, *Dicentrarchus labrax* (L.). J. Exp. Zool..

[118] Marshall W.S., Bryson S.E. (1998). Transport Mechanisms of Seawater Teleost Chloride Cells: An Inclusive Model of a Multifunctional Cell. Comp. Biochem. Physiol..

[119] Lin C.H., Tsai R.S., Lee T.H. (2004). Expression and Distribution of Na, K-ATPase in Gill and Kidney of the Spotted Green Pufferfish, Tetraodon Nigroviridis, in Response to Salinity Challenge. Comp. Biochem. Physiol. Part A.

[120] Laiz-Carrión R., Guerreiro P.M., Fuentes J., Canario A.V.M., Martín Del Río M.P., Mancera J.M. (2005). Branchial Osmoregulatory Response to Salinity in the Gilthead Sea Bream, *Sparus auratus*. J. Exp. Zool. A Comp. Exp. Biol..

[121] Prochniewicz E., Thompson L.V., Thomas D.D. (2007). Age-Related Decline in Actomyosin Structure and Function. Exp. Gerontol..

[122] Jylhävä J., Pedersen N.L., Hägg S. (2017). Biological Age Predictors. EBioMedicine.

[123] Holly A.C., Melzer D., Pilling L.C., Henley W., Hernandez D.G., Singleton A.B., Bandinelli S., Guralnik J.M., Ferrucci L., Harries L.W. (2013). Towards a Gene Expression Biomarker Set for Human Biological Age. Aging Cell.

[124] Bafei S.E.C., Shen C. (2023). Biomarkers Selection and Mathematical Modeling in Biological Age Estimation. Npj Aging.

[125] Schmieder R., Edwards R. (2011). Quality Control and Preprocessing of Metagenomic Datasets. Bioinformatics.

[126] Pérez-Sánchez J., Naya-Català F., Soriano B., Piazzon M.C., Hafez A., Gabaldón T., Llorens C., Sitjà-Bobadilla A., Calduch-Giner J.A. (2019). Genome Sequencing and Transcriptome Analysis Reveal Recent Species-Specific Gene Duplications in the Plastic Gilthead Sea Bream (*Sparus aurata*). Front. Mar. Sci..

[127] Trapnell C., Roberts A., Goff L., Pertea G., Kim D., Kelley D.R., Pimentel H., Salzberg S.L., Rinn J.L., Pachter L. (2012). Differential Gene and Transcript Expression Analysis of RNA-Seq Experiments with TopHat and Cufflinks. Nat. Protoc..

[128] Goff L., Trapnell C., Kelley D. (2013). CummeRbund: Analysis, Exploration, Manipulation, and Visualization of Cufflinks High-Throughput Sequencing Data R Package. Version 2. http://bioconductor.jp/packages/3.2/bioc/manuals/cummeRbund/man/cummeRbund.pdf.

[129] Langmead B., Trapnell C., Pop M., Salzberg S.L. (2009). Ultrafast and Memory-Efficient Alignment of Short DNA Sequences to the Human Genome. Genome Biol..

[130] Rice P., Longden I., Bleasby A. (2000). EMBOSS: The European Molecular Biology Open Software Suite. Trends Genet..

[131] Love M.I., Huber W., Anders S. (2014). Moderated Estimation of Fold Change and Dispersion for RNA-Seq Data with DESeq2. Genome Biol..

[132] Lienhard M., Grimm C., Morkel M., Herwig R., Chavez L. (2014). MEDIPS: Genome-Wide Differential Coverage Analysis of Sequencing Data Derived from DNA Enrichment Experiments. Bioinformatics.

[133] Thévenot E.A., Roux A., Xu Y., Ezan E., Junot C. (2015). Analysis of the Human Adult Urinary Metabolome Variations with Age, Body Mass Index, and Gender by Implementing a Comprehensive Workflow for Univariate and OPLS Statistical Analyses. J. Proteome Res..

[134] Weiss S., Van Treuren W., Lozupone C., Faust K., Friedman J., Deng Y., Xia L.C., Xu Z.Z., Ursell L., Alm E.J. (2016). Correlation Detection Strategies in Microbial Data Sets Vary Widely in Sensitivity and Precision. ISME J..

[135] Ge S.X., Jung D., Yao R. (2020). ShinyGO: A Graphical Gene-Set Enrichment Tool for Animals and Plants. Bioinformatics.

[136] Klopfenstein D.V., Zhang L., Pedersen B.S., Ramírez F., Vesztrocy A.W., Naldi A., Mungall C.J., Yunes J.M., Botvinnik O., Weigel M. (2018). GOATOOLS: A Python Library for Gene Ontology Analyses. Sci. Rep..

[137] Liu W., Xie Y., Ma J., Luo X., Nie P., Zuo Z., Lahrmann U., Zhao Q., Zheng Y., Zhao Y. (2015). IBS: An Illustrator for the Presentation and Visualization of Biological Sequences. Bioinformatics.

