# Supplementary material for: Exploring Multifunctional Markers of Biological Age in Farmed Gilthead Sea Bream (Sparus aurata): A Transcriptomic and Epigenetic Interplay for an Improved Fish Welfare Assessment Approach
Source: Int J Mol Sci. 2024 Sep 11;25(18):9836. doi: 10.3390/ijms25189836 (PMC11432111; doi:10.3390/ijms25189836)
Supplement: Supplementary file 1 [file ijms-25-09836-s001.zip › ijms--supplementary-proof/Figures S1 and S2 - methylome muscle.pptx]

## Slide 1
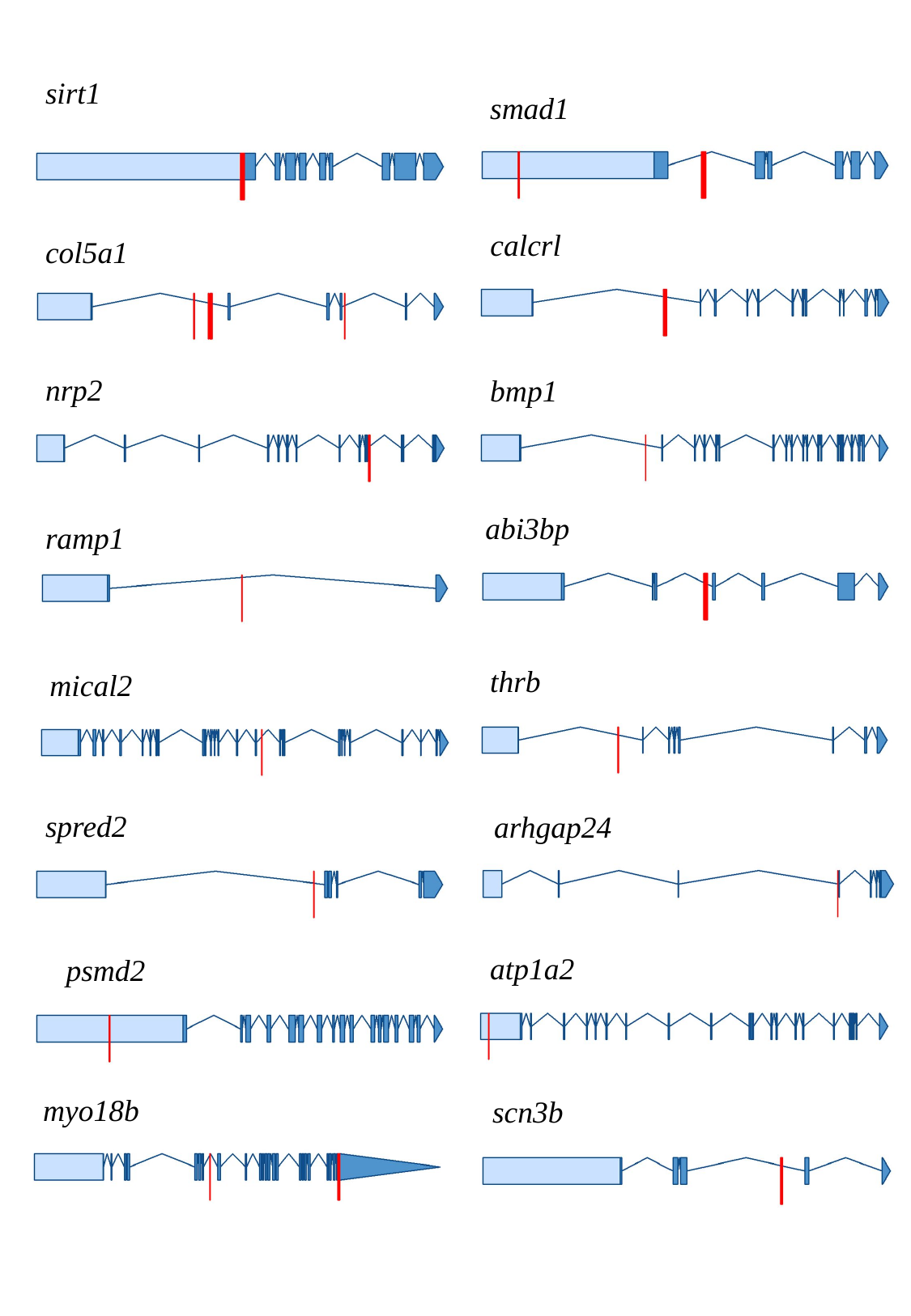

sirt1
smad1
calcrl
col5a1
nrp2
bmp1
abi3bp
ramp1
thrb
mical2
spred2
arhgap24
atp1a2
psmd2
myo18b
scn3b

## Slide 2
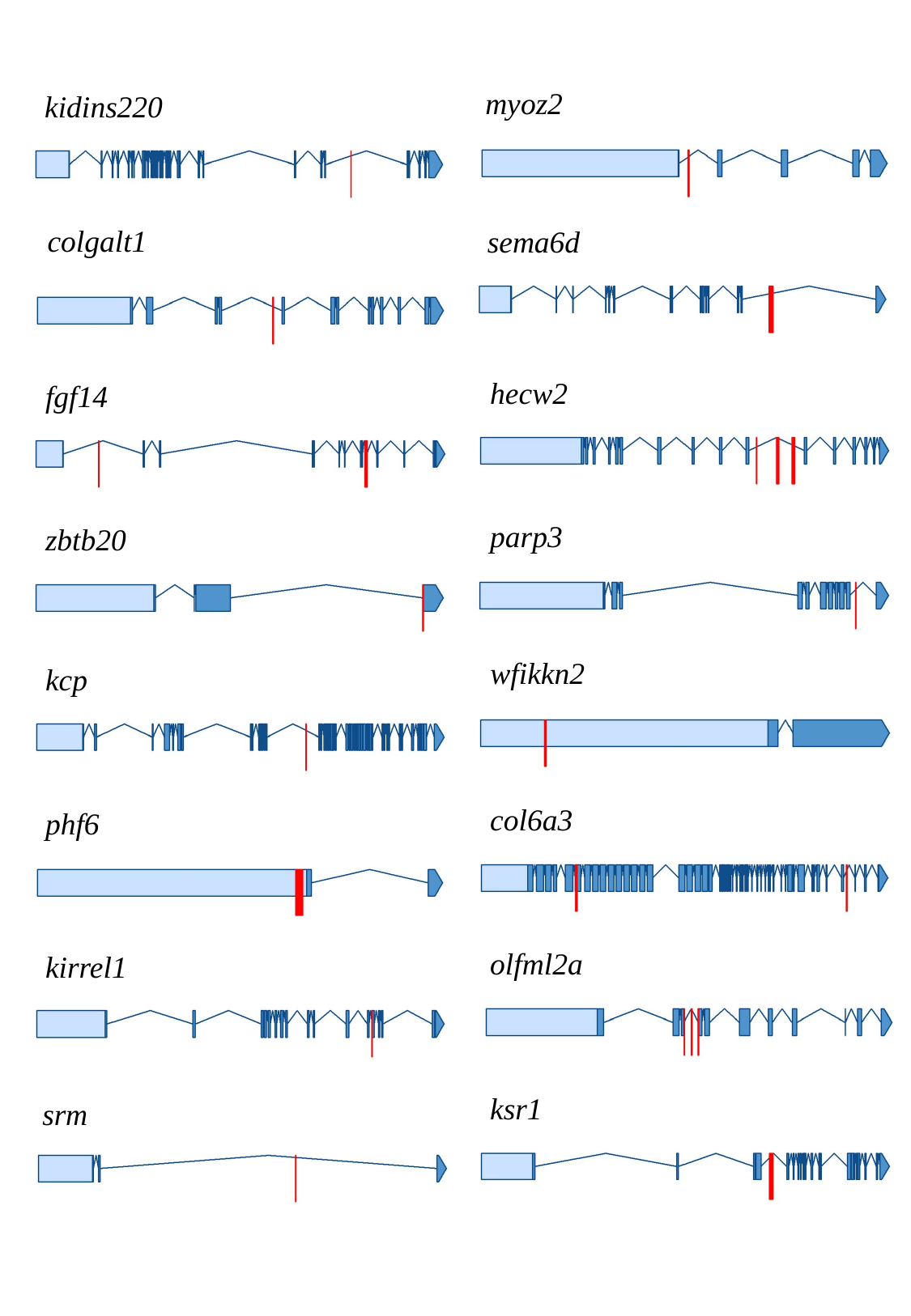

myoz2
kidins220
colgalt1
sema6d
hecw2
fgf14
parp3
zbtb20
wfikkn2
kcp
col6a3
phf6
olfml2a
kirrel1
ksr1
srm

## Slide 3
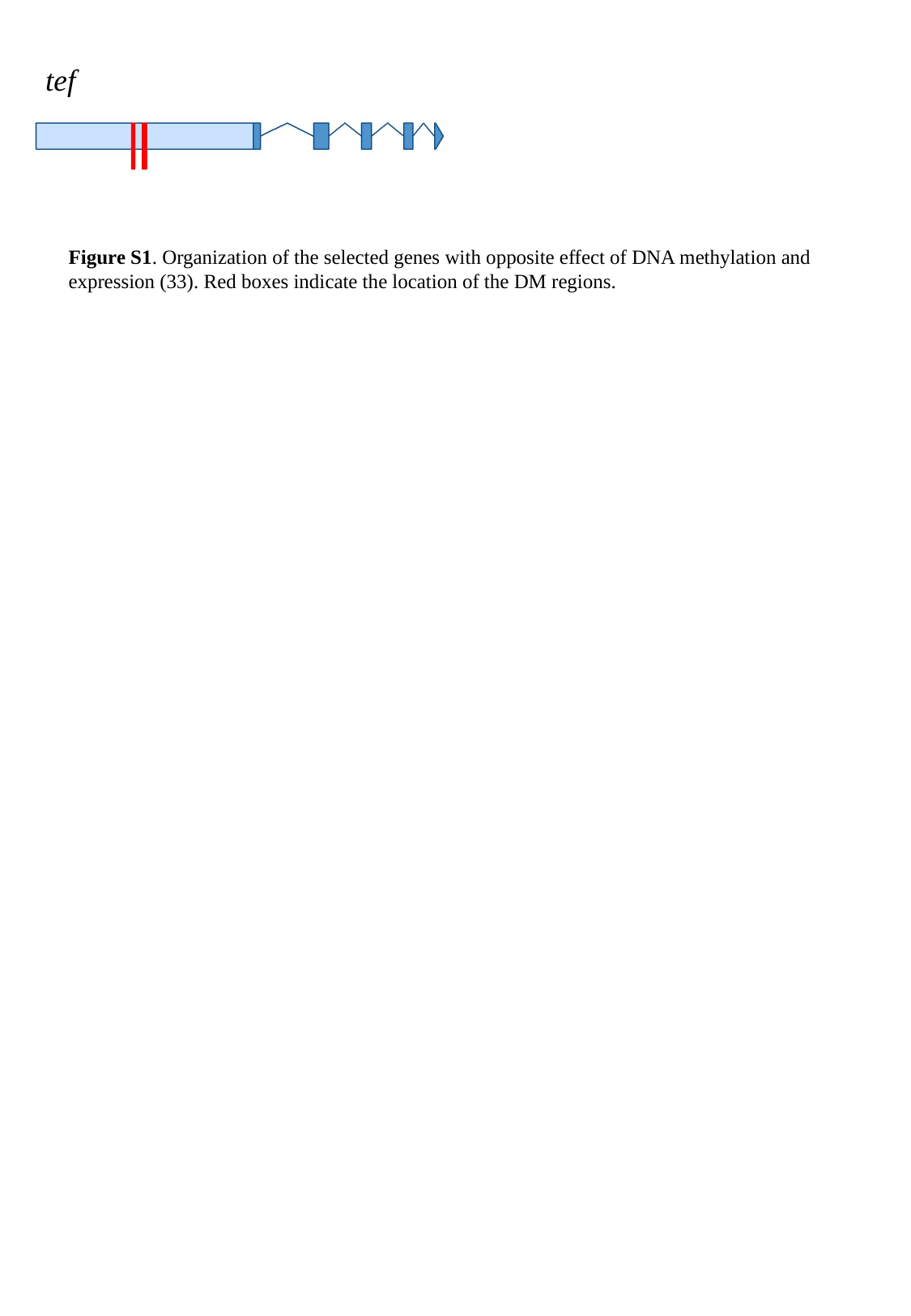

tef
Figure S1. Organization of the selected genes with opposite effect of DNA methylation and expression (33). Red boxes indicate the location of the DM regions.

## Slide 4
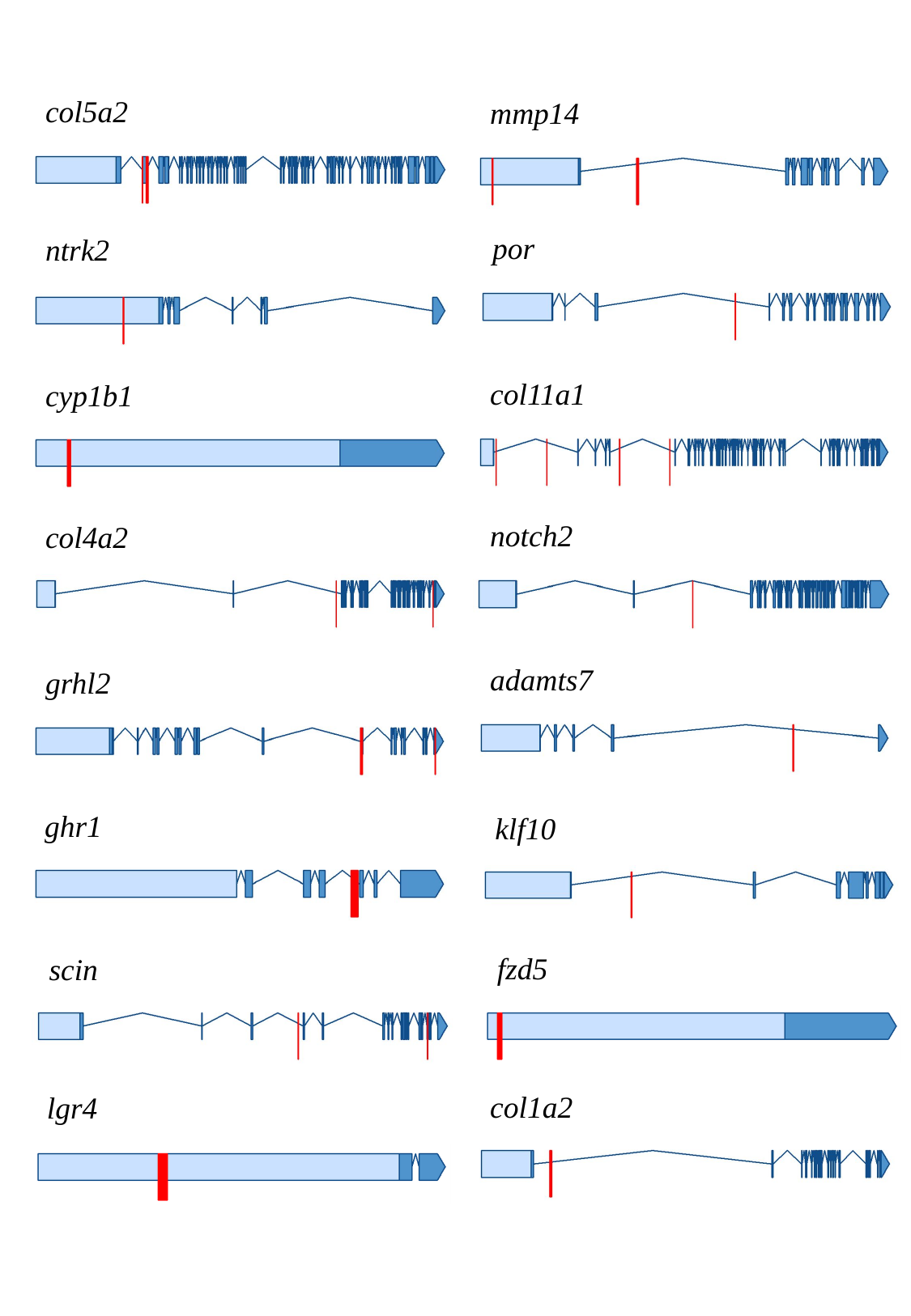

col5a2
mmp14
por
ntrk2
col11a1
cyp1b1
notch2
col4a2
adamts7
grhl2
ghr1
klf10
fzd5
scin
col1a2
lgr4

## Slide 5
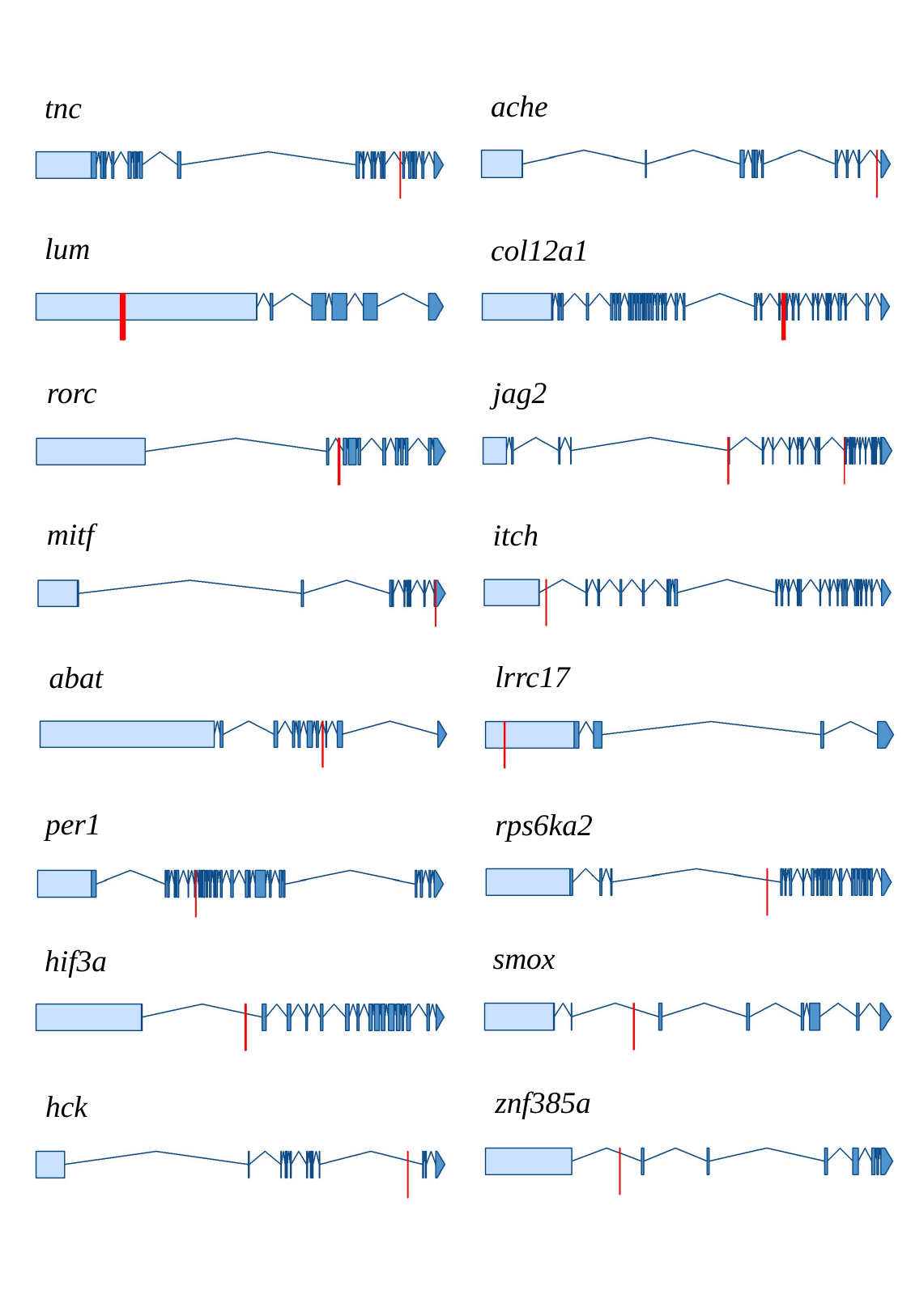

ache
tnc
lum
col12a1
rorc
jag2
mitf
itch
lrrc17
abat
per1
rps6ka2
smox
hif3a
znf385a
hck

## Slide 6
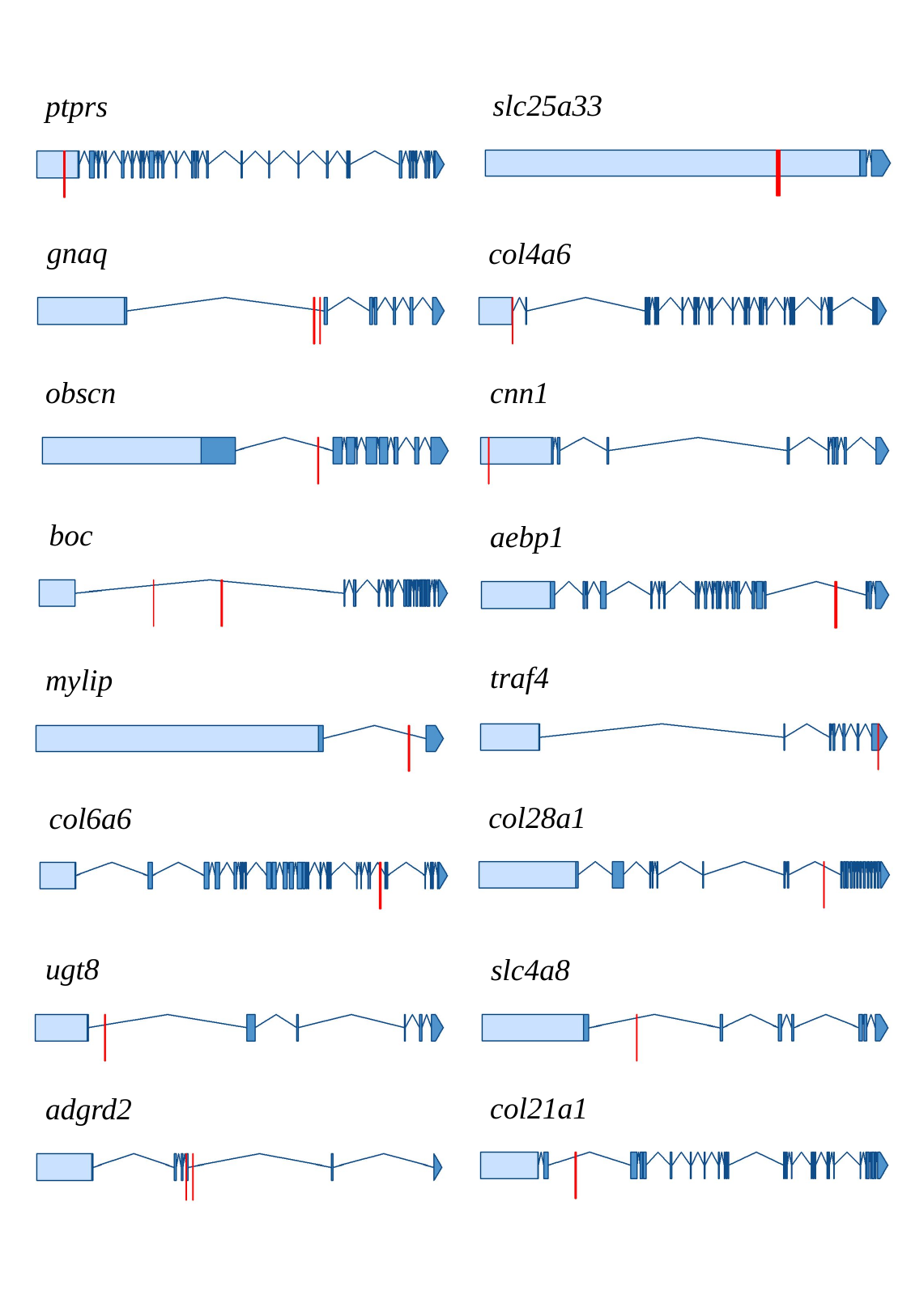

slc25a33
ptprs
gnaq
col4a6
obscn
cnn1
boc
aebp1
traf4
mylip
col28a1
col6a6
ugt8
slc4a8
col21a1
adgrd2

## Slide 7
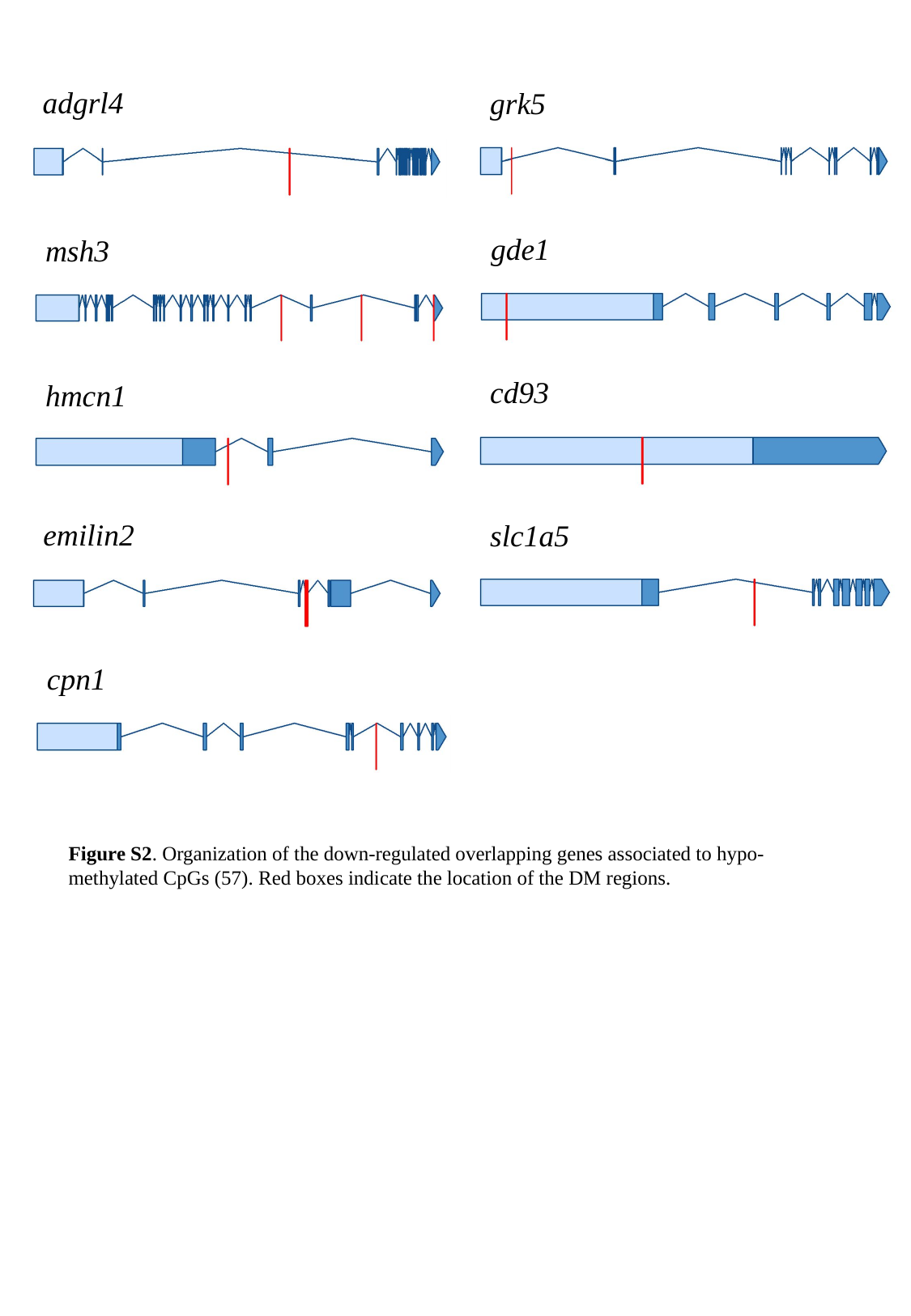

adgrl4
grk5
gde1
msh3
cd93
hmcn1
emilin2
slc1a5
cpn1
Figure S2. Organization of the down-regulated overlapping genes associated to hypo-methylated CpGs (57). Red boxes indicate the location of the DM regions.
