# Supplementary material for: Exploring Multifunctional Markers of Biological Age in Farmed Gilthead Sea Bream (Sparus aurata): A Transcriptomic and Epigenetic Interplay for an Improved Fish Welfare Assessment Approach
Source: Int J Mol Sci. 2024 Sep 11;25(18):9836. doi: 10.3390/ijms25189836 (PMC11432111; doi:10.3390/ijms25189836)
Supplement: Supplementary file 1 [file ijms-25-09836-s001.zip › ijms--supplementary-proof/Table S1. Detailed sequencing metrics.docx]

| **Experiment** | **Sample ID** | **Raw Reads** | **Pre-Processed Reads**  **(%)** | **Mapped Reads**  **(%)** | **Saturation** |
| --- | --- | --- | --- | --- | --- |
| **MBD-Seq** | DNA_S1_1 | 42,639,544 | 41,924,296 (98.32) | 40,611,211 (96.87) | 0.99 |
|  | DNA_S1_2 | 42,552,229 | 41,882,858 (98.43) | 40,589,822 (96.91) | 0.99 |
|  | DNA_S1_3 | 46,466,064 | 45,744,958 (98.45) | 44,338,251 (96.92) | 0.99 |
|  | DNA_S1_4 | 35,100,171 | 34,453,921 (98.16) | 33,420,480 (97.01) | 0.99 |
|  | DNA_S1_5 | 43,065,143 | 42,393,949 (98.44) | 41,101,727 (96.95) | 0.98 |
|  | DNA_S1_6 | 43,013,078 | 42,287,628 (98.31) | 41,007,744 (96.97) | 0.98 |
|  | DNA_S1_7 | 46,176,858 | 45,546,930 (98.64) | 44,220,943 (97.09) | 0.99 |
|  | DNA_S3_1 | 41,530,064 | 40,877,297 (98.43) | 39,372,665 (96.32) | 0.99 |
|  | DNA_S3_2 | 44,338,065 | 43,408,998 (97.90) | 41,968,048 (96.68) | 0.99 |
|  | DNA_S3_3 | 33,466,135 | 33,036,141 (98.72) | 32,060,293 (97.05) | 0.99 |
|  | DNA_S3_4 | 33,696,616 | 33,229,905 (98.61) | 32,207,766 (96.92) | 0.98 |
|  | DNA_S3_5 | 35,811,847 | 35,181,488 (98.24) | 34,100,754 (96.93) | 0.98 |
|  | DNA_S3_6 | 35,469,514 | 34,756,609 (97.99) | 33,512,134 (96.42) | 0.98 |
|  | DNA_S3_7 | 44,740,740 | 43,948,478 (98.23) | 42,041,126 (95.66) | 0.98 |
|  |  |  |  |  |  |
| **RNA-seq** | RNA_S1_1 | 35,648,711 | 35,589,123 (99.83) | 31,709,909 (89.1) |  |
|  | RNA_S1_2 | 46,976,011 | 46,896,803 (99.83) | 42,019,535 (89.6) |  |
|  | RNA_S1_3 | 41,600,604 | 41,524,179 (99.82) | 37,039,568 (89.2) |  |
|  | RNA_S1_4 | 44,870,694 | 44,797,248 (99.84) | 40,183,131 (89.7) |  |
|  | RNA_S1_5 | 30,792,262 | 30,228,343 (98.17) | 26,933,454 (89.1) |  |
|  | RNA_S1_6 | 36,901,501 | 36,816,181 (99.77) | 33,097,747 (89.9) |  |
|  | RNA_S1_7 | 32,857,206 | 32,773,289 (99.74) | 29,430,414 (89.8) |  |
|  | RNA_S3_1 | 31,649,656 | 31,549,846 (99.68) | 28,426,411 (90.1) |  |
|  | RNA_S3_2 | 32,480,074 | 32,439,715 (99.88) | 28,741,587 (88.6) |  |
|  | RNA_S3_3 | 40,629,793 | 40,563,421 (99.84) | 36,466,515 (89.9) |  |
|  | RNA_S3_4 | 34,229,322 | 33,884,316 (98.99) | 30,766,959 (90.8) |  |
|  | RNA_S3_5 | 43,128,031 | 42,729,255 (99.08) | 39,011,810 (91.3) |  |
|  | RNA_S3_6 | 42,480,807 | 42,416,050 (99.85) | 38,513,773 (90.8) |  |
|  | RNA_S3_7 | 31,547,703 | 31,475,021 (99.77) | 28,831,119 (91.6) |  |

**Table S1**. Detailed sequencing data obtained in this study.
