# Supplementary material for: Exploring Multifunctional Markers of Biological Age in Farmed Gilthead Sea Bream (Sparus aurata): A Transcriptomic and Epigenetic Interplay for an Improved Fish Welfare Assessment Approach
Source: Int J Mol Sci. 2024 Sep 11;25(18):9836. doi: 10.3390/ijms25189836 (PMC11432111; doi:10.3390/ijms25189836)
Supplement: Supplementary file 1 [file ijms-25-09836-s001.zip › ijms--supplementary-proof/Table S2. Real-time PCR validation.docx]

Supplementary Table S2. Comparison between RNA-seq results and real-time PCR validation. Values are the mean ± SEM of seven samples per age class.

| **Fold change (S+3/S+1)** | | | | |
| --- | --- | --- | --- | --- |
| **Gene Name** | **Symbol** | **GenBank**  **accession** | **RNA-seq** | **Real-time PCR** |
| Calpain-1 catalytic subunit | *capn1* | KF444899 | -2.46 ± 0.32 | -1.59 ± 0.12 |
| Carnitine palmitoyltransferase 1A | *cpt1a* | JQ308822 | -2.86 ± 0.38 | -2.65 ± 0.25 |
| CD209 antigen-like protein D | *cd209d* | KF857327 | -2.39 ± 0.42 | -1.77 ± 0.16 |
| Diacylglycerol O-acyltransferase 2 | *dgat2* | MG570177 | 2.58 ± 0.49 | 2.85 ± 0.27 |
| Intestinal fatty acid-binding protein | *fabp2* | KF857310 | 3.16 ± 0.69 | 3.93 ± 0.33 |
| Macrophage mannose receptor 1 | *mrc1* | KF857326 | -2.32 ± 0.38 | -1.67 ± 0.11 |
| NAD-dependent deacetylase sirtuin-1 | *sirt1* | KF018666 | 2.15 ± 0.23 | 1.87 ± 0.10 |
| Uncoupling protein 3 | *ucp3* | EU555336 | -2.06 ± 0.17 | -2.08 ± 0.21 |
