# Supplementary material for: Exploring Multifunctional Markers of Biological Age in Farmed Gilthead Sea Bream (Sparus aurata): A Transcriptomic and Epigenetic Interplay for an Improved Fish Welfare Assessment Approach
Source: Int J Mol Sci. 2024 Sep 11;25(18):9836. doi: 10.3390/ijms25189836 (PMC11432111; doi:10.3390/ijms25189836)
Supplement: Supplementary file 1 [file ijms-25-09836-s001.zip › ijms--supplementary-proof/Tables S4 and S5 - methylome muscle.docx]

**Table S4.** Differential expression (log_2_ fold-change S+3 vs S+1, log_2_ FC (exp)) and location (scaffold and chromosome, chr) of the selected genes with opposite effect of methylation and expression (potential epigenetic markers) and their corresponding putative differentially methylated (DM) regions. The genomic area (Feature: promoter, Prom, exon or intron) where these DM regions are positioned and their differential methylation (log_2_ fold-change S+3 vs S+1, log_2_ FC (met)) and the number of CpGs are also shown. Genes in red indicates up-regulation and in bold means that are located in a regulating area (Prom, Exon 1, Intron 1).

| **Gene** | **Log_2_ FC (exp)** | **Scaffold (chr)** | **Position**  **DM region** | **Feature** | **Log_2_ FC (met)** | **Nº CpG** |
| --- | --- | --- | --- | --- | --- | --- |
| **NAD-dependent deacetylase sirtuin-1 (*sirt1*)** | 1,10 | 3643 | 8560276-8560300 | Prom | -1,51 | 1 |
|  |  |  | 8560301-8560325 | Exon 1 | -1,23 | 3 |
|  |  |  | 8560326-8560350 | Exon 1 | -1,26 | 2 |
|  |  |  | 8560351-8560375 | Exon 1 | -1,23 | 3 |
|  |  |  |  |  |  |  |
| **Mothers against decapentaplegic homolog 5 (*smad1*)** | 0,58 | 3457 | 126451-126475 | Intron 1 | -2,12 | 1 |
|  |  |  | 126551-126575 | Intron 1 | -1,84 | 1 |
|  |  |  | 131876-131900 | Prom | -2,22 | 1 |
|  |  |  |  |  |  |  |
| **Collagen alpha-1(V) chain (*col5a1*)** | -0,98 | 2979 | 307376-307400 | Intron 4 | 2,11 | 1 |
|  |  |  | 319801-319825 | Intron 1 | 1,72 | 1 |
|  |  |  | 319826-319850 | Intron 1 | 2,12 | 2 |
|  |  |  | 319851-319875 | Intron 1 | 2,13 | 1 |
|  |  |  | 319876-319900 | Intron 1 | 2,13 | 1 |
|  |  |  | 319901-319925 | Intron 1 | 1,89 | 2 |
|  |  |  | 319926-319950 | Intron 1 | 1,17 | 1 |
|  |  |  | 319951-319975 | Intron 1 | 1,12 | 2 |
|  |  |  | 319976-320000 | Intron 1 | 1,06 | 4 |
|  |  |  | 320126-320150 | Intron 1 | 1,30 | 1 |
|  |  |  | 321451-321475 | Intron 1 | 1,44 | 3 |
|  |  |  | 321476-321500 | Intron 1 | 1,50 | 3 |
|  |  |  | 321501-321525 | Intron 1 | 1,50 | 3 |
|  |  |  |  |  |  |  |
| **Calcitonin gene-related peptide type 1 receptor-like (*calcrl*)** | -0,75 | 3563 | 1773826-1773850 | Intron 1 | 1,11 | 4 |
|  |  |  | 1773901-1773925 | Intron 1 | 1,06 | 2 |
|  |  |  | 1773926-1773950 | Intron 1 | 1,04 | 2 |
|  |  |  | 1773951-1773975 | Intron 1 | 1,24 | 1 |
|  |  |  | 1774026-1774050 | Intron 1 | 0,93 | 1 |
|  |  |  | 1774076-1774100 | Intron 1 | 0,97 | 3 |
|  |  |  | 1774101-1774125 | Intron 1 | 1,04 | 2 |
|  |  |  | 1774126-1774150 | Intron 1 | 1,06 | 1 |
|  |  |  |  |  |  |  |
| Neuropilin-2 (*nrp2*) | -0,83 | 3494 | 174451-174475 | Intron 12 | 2,15 | 3 |
|  |  |  | 174476-174500 | Intron 12 | 2,24 | 2 |
|  |  |  | 174501-174525 | Intron 12 | 2,24 | 4 |
|  |  |  | 174601-174625 | Intron 12 | 1,40 | 1 |
|  |  |  | 174626-174650 | Intron 12 | 1,38 | 4 |
|  |  |  | 174651-174675 | Intron 12 | 1,38 | 4 |
|  |  |  | 174676-174680 | Intron 12 | 1,38 | 1 |
|  |  |  | 174701-174725 | Intron 12 | 1,38 | 3 |
|  |  |  | 174726-174750 | Intron 12 | 1,38 | 3 |
|  |  |  |  |  |  |  |
| **Bone morphogenetic protein 1 (*bmp1*)** | -1,49 | 3629 | 1809026-1809050 | Intron 1 | 1,82 | 1 |
|  |  |  |  |  |  |  |
| **Receptor activity-modifying protein 1 (*ramp1*)** | 1,61 | 3252 | 210176-210200 | Intron 1 | -1,43 | 1 |
|  |  |  |  |  |  |  |
| Target of Nesh-SH3 (*abi3bp*) | -1,33 | 2739 | 43851-43875 | Intron 3 | 1,44 | 1 |
|  |  |  | 43876-43900 | Intron 3 | 1,44 | 1 |
|  |  |  | 43926-43950 | Intron 3 | 1,52 | 2 |
|  |  |  | 43976-44000 | Intron 3 | 1,27 | 1 |
|  |  |  | 44026-44050 | Intron 3 | 1,27 | 2 |
|  |  |  | 44051-44076 | Intron 3 | 1,27 | 1 |
|  |  |  |  |  |  |  |
| Protein MICAL-2 (*mical2*) | -0,64 | 3177 | 465101-465125 | Intron 15 | 0,90 | 1 |
|  |  |  | 465126-465150 | Intron 15 | 0,96 | 1 |
|  |  |  |  |  |  |  |
| **Thyroid hormone receptor beta (*thrb*)** | -1,36 | 3101 | 60076-60100 | Intron 1 | 2,05 | 1 |
|  |  |  | 60101-60125 | Intron 1 | 2,05 | 3 |
|  |  |  | 60126-60150 | Intron 1 | 1,86 | 2 |
|  |  |  | 60151-60175 | Intron 1 | 1,70 | 3 |
|  |  |  |  |  |  |  |
| **Sprouty-related, EVH1 domain-containing protein 2 (*spred2*)** | -0,92 | 3097 | 442351-442375 | Intron 1 | * | 1 |
|  |  |  |  |  |  |  |
| Rho GTPase-activating protein 24 (*arhgap24*) | -0,82 | 3620 | 4065201-4065225 | Intron 3 | 2,59 | 1 |
|  |  |  |  |  |  |  |
| **26S proteasome non-ATPase regulatory subunit 2 (*psmd2*)** | 0,97 | 101 | 1751-1775 | Prom | -2,81 | 1 |
|  |  |  |  |  |  |  |
| **Sodium/potassium-transporting ATPase subunit alpha-2 (*atp1a2*)** | -0,67 | 3644 | 3109626-3109650 | Prom | 1,37 | 2 |
|  |  |  | 3109651-3109675 | Prom | 1,24 | 1 |
|  |  |  |  |  |  |  |
| Unconventional myosin-XVIIIb-like (*myo18b*) | -0,74 | 1719 | 76576-76600 | Exon 24 | 1,32 | 1 |
|  |  |  | 76601-76625 | Exon 24 / Intron 23 | 1,39 | 1 |
|  |  |  | 76626-76650 | Intron 23 | 1,45 | 1 |
|  |  |  | 76651-76675 | Intron 23 | 1,56 | 1 |
|  |  |  | 76676-76700 | Exon 23 / Intron 23 | 1,56 | 1 |
|  |  |  | 86001-86025 | Intron 7 | 1,49 | 1 |
|  |  |  |  |  |  |  |
| Sodium channel subunit beta-3 (*scn3b*) | 1,51 | 3552 | 1471551-1471575 | Intron 3 | -1,13 | 1 |
|  |  |  | 1471601-1471625 | Intron 3 | -1,08 | 1 |
|  |  |  |  |  |  |  |
| Kinase D-interacting substrate of 220 kDa (*kidins220*) | -0,61 | 3646 | 7083126-7083150 | Intron 25 | 2,31 | 1 |
|  |  |  |  |  |  |  |
| **Myozenin-2-like isoform X1 (*myoz2*)** | 1,53 | 3415 | 622651-622675 | Intron 1 | -1,14 | 1 |
|  |  |  |  |  |  |  |
| Procollagen galactosyltransferase 1 (*colgalt1*) | -1,13 | 3579 | 2011901-2011925 | Intron 4 | 3,16 | 1 |
|  |  |  |  |  |  |  |
| Semaphorin-6D (*sema6d*) | -0,87 | 2966 | 235876-235900 | Intron 14 | 1,36 | 2 |
|  |  |  | 235951-235975 | Intron 14 | 1,75 | 1 |
|  |  |  | 235976-236000 | Intron 14 | 1,85 | 1 |
|  |  |  | 236001-236025 | Intron 14 | 1,96 | 2 |
|  |  |  | 236026-236050 | Intron 14 | 2,01 | 1 |
|  |  |  | 236051-236075 | Intron 14 | 2,01 | 2 |
|  |  |  | 236076-236100 | Intron 14 | 1,82 | 1 |
|  |  |  | 236151-236175 | Intron 14 | 1,66 | 1 |
|  |  |  | 236176-236200 | Intron 14 | 1,05 | 2 |
|  |  |  | 236201-236225 | Intron 14 | 0,99 | 1 |
|  |  |  | 236251-236275 | Intron 14 | 1,02 | 2 |
|  |  |  | 236276-236300 | Intron 14 | 0,85 | 1 |
|  |  |  | 236301-236325 | Intron 14 | 1,01 | 1 |
|  |  |  | 236376-236400 | Intron 14 | 1,06 | 1 |
|  |  |  | 236401-236425 | Intron 14 | 0,97 | 2 |
|  |  |  | 236451-236475 | Intron 14 | 0,97 | 2 |
|  |  |  |  |  |  |  |
| Fibroblast growth factor 14 (*fgf14*) | -0,58 | 2952 | 80726-80750 | Intron 10 | 1,22 | 2 |
|  |  |  | 80876-80900 | Exon 10 | 1,21 | 1 |
|  |  |  | 80901-80925 | Exon 10 | 1,06 | 1 |
|  |  |  | 80926-80950 | Exon 10 | 0,97 | 2 |
|  |  |  | 81026-81050 | Intron 9 | 0,98 | 2 |
|  |  |  | 81101-81125 | Intron 9 | 1,09 | 2 |
|  |  |  | 130901-130925 | Intron 1 | 0,72 | 1 |
|  |  |  | 130926-130950 | Intron 1 | 0,78 | 2 |
|  |  |  | 130951-.130975 | Intron 1 | 0,84 | 2 |
|  |  |  | 130976-131000 | Intron 1 | 0,80 | 1 |
|  |  |  | 131001-131025 | Intron 1 | 0,75 | 1 |
|  |  |  |  |  |  |  |
| E3 ubiquitin-protein ligase (*hecw2*) | -1.22 | 1137 | 41051-41075 | Intron 10 | 1,31 | 3 |
|  |  |  | 41076-41100 | Intron 10 | 1,32 | 2 |
|  |  |  | 41126-41150 | Intron10 | 1,38 | 1 |
|  |  |  | 41151-41175 | Intron 10 | 1,38 | 2 |
|  |  |  | 41826-41850 | Intron 10 | 2,77 | 4 |
|  |  |  | 41851-41875 | Intron 10 | 2,77 | 2 |
|  |  |  | 41901-41925 | Intron 10 | 2,77 | 3 |
|  |  |  | 42926-41950 | Intron 10 | 0,77 | 4 |
|  |  |  |  |  |  |  |
| Zinc finger and BTB domain-containing protein 20 (*zbtb20*) | -0,89 | 28 | 8730351-8730375 | Exon 4 | 1,58 | 2 |
|  |  |  |  |  |  |  |
| Poly [ADP-ribose] polymerase 3 (*parp3*) | 0,72 | 3609 | 1672851-1672875 | Intron 10 | -1,48 | 1 |
|  |  |  |  |  |  |  |
| Kielin/chordin-like protein (*kcp*) | -0,85 | 3614 | 4005251-4005275 | Intron 14 | 1,91 | 1 |
|  |  |  |  |  |  |  |
| **WAP, kazal, immunoglobulin, kunitz and NTR domain-containing protein 2 (*wfikkn2*)** | -1.28 | 3645 | 8288601-8288625 | Prom | 0.98 | 2 |
|  |  |  |  |  |  |  |
| **PHD finger protein 6 isoform X1 (*phf6*)** | 1.30 | 3619 | 4170576-4170600 | Prom | -1,75 | 1 |
|  |  |  | 4170601-4170625 | Prom | -1,75 | 1 |
|  |  |  | 4170626-4170650 | Prom | -1,71 | 1 |
|  |  |  | 4170651-4170675 | Prom | -1,71 | 2 |
|  |  |  | 4170676-4170680 | Prom | -1,47 | 1 |
|  |  |  |  |  |  |  |
| Collagen alpha-3(VI) chain (*col6a3*) | -0,70 | 2388 | 6326-6350 | Exon 6 | 0,72 | 2 |
|  |  |  | 6376-6400 | Exon 6 | 1,17 | 2 |
|  |  |  | 6401-6425 | Exon 6 | 1,17 | 1 |
|  |  |  | 6526-6550 | Exon 6 | 0,84 | 3 |
|  |  |  | 6551-6575 | Exon 6 | 0,91 | 1 |
|  |  |  | 6876-6900 | Intron 52 | 2,38 | 1 |
|  |  |  | 35601-35625 | Intron 52 | 1,62 | 1 |
|  |  |  | 35626-35650 | Intron 52 | 1,51 | 2 |
|  |  |  | 35651-35675 | Intron 52 | 1,51 | 2 |
|  |  |  |  |  |  |  |
| Kin of IRRE-like protein 1 isoform X2 (*kirrel1*) | -0,78 | 1921 | 59376-59380 | Intron 12 | 1,06 | 1 |
|  |  |  |  |  |  |  |
| Olfactomedin-like protein 2ª (*olfml2a*) | -1,31 | 2720 | 51176-51200 | Intron 3 | 0,73 | 3 |
|  |  |  | 51501-51525 | Intron 3 | 0,97 | 1 |
|  |  |  | 51801-51825 | Exon 4 / Intron 3 | 3,36 | 1 |
|  |  |  |  |  |  |  |
| Spermidine synthase (*srm*) | 1,15 | 3600 | 2248001-2248025 | Intron 2 | -3,17 | 1 |
|  |  |  |  |  |  |  |
| Kinase suppressor of Ras 1 (*ksr1*) | -1,01 | 3405 | 328301-328352 | Intron 4 | 0,67 | 1 |
|  |  |  | 328401-328425 | Intron 4 | 0,83 | 2 |
|  |  |  | 328426-328450 | Intron 4 | 0,94 | 1 |
|  |  |  | 328451-328475 | Intron 4 | 1,00 | 1 |
|  |  |  | 328476-328500 | Intron 4 | 1,00 | 1 |
|  |  |  | 328501-328525 | Intron 4 | 1,02 | 3 |
|  |  |  | 328526-328550 | Intron 4 | 1,01 | 1 |
|  |  |  | 328551-328575 | Intron 4 | 0,98 | 1 |
|  |  |  | 328576-328600 | Intron 4 | 0,98 | 1 |
|  |  |  | 328601-328625 | Intron 4 | 0,98 | 1 |
|  |  |  |  |  |  |  |
| **Thyrotroph embryonic factor (*tef*)** | -0,86 | 3645 | 4166926-4166950 | Prom | 1,95 | 1 |
|  |  |  | 4166951-9166975 | Prom | 1,70 | 1 |
|  |  |  | 4166976-4167000 | Prom | 1,70 | 2 |
|  |  |  | 4167001-4167025 | Prom | 1,70 | 1 |
|  |  |  | 4167201-4167225 | Prom | 1,54 | 1 |
|  |  |  | 4167251-4167275 | Prom | 1,48 | 1 |

**Table S5.** Differential expression (log_2_ fold-change S+3 vs S+1, log_2_ FC (exp)) and location (scaffold and chromosome, chr) of the selected genes with similar effect of methylation and expression (potential epigenetic markers) and their corresponding putative differentially methylated (DM) regions. The genomic area (Feature: promoter, Prom, exon or intron) where these DM regions are positioned and their differential methylation (log_2_ fold-change S+3 vs S+1, log_2_ FC (met)) and the number of CpGs are also shown. Genes in bold means that are located in a regulating area (Prom, Exon 1, Intron 1).

| **Gene** | **Log_2_ FC (exp)** | **Scaffold (chr)** | **Position**  **DM region** | **Feature** | **Log_2_ FC (met)** | **Nº CpG** |
| --- | --- | --- | --- | --- | --- | --- |
| Collagen alpha-2(V) chain (*col5a2*) | -1.74 | 2983 | 226576-226600 | Exon 2 / Intron 1 | -0.95 | 1 |
|  |  |  | 226826226850 | Exon 2 | -3.11 | 1 |
|  |  |  | 226926-226950 | Intron 2 | -3.12 | 2 |
|  |  |  |  |  |  |  |
| **Matrix metalloproteinase-14 (*mmp14*)** | -0.75 | 3491 | 1072451-1072475 | Intron 1 | -1.71 | 1 |
|  |  |  | 1072526-1072550 | Intron 1 | -1.80 | 1 |
|  |  |  | 1079851-1079875 | Prom | -1.75 | 2 |
|  |  |  | 1079876-1079900 | Prom | -1.75 | 1 |
|  |  |  |  |  |  |  |
| **BDNF/NT-3 growth factors receptor isoform X4 (*ntrk2*)** | -2.04 | 2980 | 253076-253100 | Prom | -0.86 | 1 |
|  |  |  |  |  |  |  |
| NADPH--cytochrome P450 reductase (Fragments) (*por*) | -0.69 | 3617 | 3875351-3875375 | Intron 3 | -1.39 | 2 |
|  |  |  |  |  |  |  |
| **Cytochrome P450 1B1 (*cyp1b1*)** | -0.67 | 3646 | 4523451-4523475 | Prom | -1.25 | 1 |
|  |  |  | 4523476-4523500 | Prom | -1.25 | 1 |
|  |  |  |  |  |  |  |
| Collagen alpha-1(XI) chain (*col11a1*) | -1.41 | 3508 | 901701-901725 | Intron 5 | -0.77 | 1 |
|  |  |  | 901726-901750 | Intron 5 | -0.80 | 1 |
|  |  |  | 921426-921450 | Intron 5 | -1.72 | 1 |
|  |  |  | 921476-921500 | Intron 5 | -1.72 | 1 |
|  |  |  | 921576-921600 | Intron 5 | -2.53 | 2 |
|  |  |  | 950251-950275 | Intron 1 | -0.95 | 1 |
|  |  |  | 970176-970200 | Intron 1 | -1.15 | 1 |
|  |  |  | 970201-970225 | Intron 1 | -1.26 | 1 |
|  |  |  |  |  |  |  |
| Collagen alpha-2(IV) chain (Fragment) (*col4a2*) | -0.81 | 3558 | 1927451-1927475 | Intron 3 | -1.61 | 1 |
|  |  |  | 1954176-1954200 | Intron 46 | -1.63 | 1 |
|  |  |  |  |  |  |  |
| Neurogenic locus notch homolog protein 2 (*notch2*) | -0.77 | 3638 | 2111151-2111175 | Intron 2 | -1.74 | 1 |
|  |  |  |  |  |  |  |
| Grainyhead-like protein 2 homolog (*grhl2*) | -2.42 | 3296 | 272051-272075 | Exon 18 | -1.05 | 3 |
|  |  |  | 272076-272100 | Exon 18 | -1.05 | 3 |
|  |  |  | 277051-277075 | Exon 11 | -1.61 | 1 |
|  |  |  | 277076-277100 | Exon 11 | -1.61 | 3 |
|  |  |  | 277151-277175 | Exon 11 | -1.61 | 1 |
|  |  |  |  |  |  |  |
| A disintegrin and metalloproteinase with thrombospondin motifs 7-like (*adamts7*) | -1.31 | 2281 | 12426-12450 | Intron 4 | -2.21 | 1 |
|  |  |  | 12451-12475 | Intron 4 | -2.21 | 1 |
|  |  |  |  |  |  |  |
| Growth hormone receptor type I (*ghr1*) | -0.86 | 4678 | 2851-2875 | Intron 4 | -2.18 | 1 |
|  |  |  | 3001-3025 | Intron 4 | -1.52 | 1 |
|  |  |  |  |  |  |  |
| **Krueppel-like factor 10 (*klf10*)** | -0.68 | 3148 | 415451-415475 | Intron 1 | -2.13 | 2 |
|  |  |  | 415476-415500 | Intron 1 | -2.13 | 2 |
|  |  |  |  |  |  |  |
| Adseverin (*scin*) | -0.65 | 3644 | 4998601-4998625 | Exon 15 | -2.01 | 1 |
|  |  |  | 5014176-5014200 | Intron 3 | -1.44 | 4 |
|  |  |  |  |  |  |  |
| **Frizzled-5 (*fzd5*)** | -1.80 | 2609 | 217126-217150 | Prom | -1.12 | 2 |
|  |  |  | 217176-217200 | Prom | -1.12 | 1 |
|  |  |  |  |  |  |  |
| **Leucine-rich repeat-containing G-protein coupled receptor 4 (*lgr4*)** | -1.37 | 3574 | 861551-861575 | Prom | -1.55 | 1 |
|  |  |  | 861601-861625 | Prom | -1.62 | 2 |
|  |  |  | 861651-861675 | Prom | -1.70 | 1 |
|  |  |  |  |  |  |  |
| **Collagen alpha-2(I) chain precursor (*col1a2*)** | -1.13 | 2646 | 96551-96575 | Intron 1 | -1.85 | 1 |
|  |  |  | 96576-96600 | Intron 1 | -1.85 | 2 |
|  |  |  | 96601-96625 | Intron 1 | -1.85 | 2 |
|  |  |  | 96626-96650 | Intron 1 | -1.75 | 2 |
|  |  |  | 96701-96725 | Intron 1 | -1.75 | 1 |
|  |  |  | 96726-96750 | Intron 1 | -1.75 | 2 |
|  |  |  |  |  |  |  |
| Tenascin (*tnc*) | -0.87 | 3244 | 480901-480925 | Intron 15 | -1.30 | 1 |
|  |  |  |  |  |  |  |
| Acetylcholinesterase (*ache*) | -0.80 | 3417 | 279376-279400 | Intron 9 | -1.07 | 1 |
|  |  |  |  |  |  |  |
| **Lumican-like (*lum*)** | -1.36 | 3582 | 1376751-1376775 | Prom | -1.05 | 1 |
|  |  |  | 1376776-1376800 | Prom | -1.05 | 1 |
|  |  |  | 1376826-1376850 | Prom | -1.19 | 1 |
|  |  |  |  |  |  |  |
| Collagen alpha-1(XII) chain-like, partial (*col12a1*) | -1.35 | 3370 | 580926-580950 | Intron 13 | -1.62 | 1 |
|  |  |  | 602126-602150 | Intron 5 | -1.16 | 1 |
|  |  |  | 602151-602175 | Intron 5 | -1.16 | 2 |
|  |  |  |  |  |  |  |
| Nuclear receptor ROR-gamma (*rorc*) | -0.85 | 3601 | 1951876-1951900 | Intron 2 | -2.29 | 1 |
|  |  |  | 1951926-1951950 | Intron 2 | -1.39 | 1 |
|  |  |  |  |  |  |  |
| Protein jagged-2-like isoform X1 (*jag2*) | -0.71 | 3527 | 1529376-1529400 | Exon 15 | -0.82 | 1 |
|  |  |  | 1554076-1554100 | Intron 4 | -1.63 | 1 |
|  |  |  | 1554151-1554175 | Intron 4 | -1.86 | 1 |
|  |  |  | 1554176-1554200 | Intron 4 | -1.86 | 1 |
|  |  |  |  |  |  |  |
| microphthalmia-associated transcription factor-like (*mitf*) | -0.88 | 3455 | 547226-547250 | Exon 10 | -1.27 | 2 |
|  |  |  | 547251-547275 | Exon 10 | -1.27 | 2 |
|  |  |  | 547276-547300 | Exon 10 | -1.27 | 1 |
|  |  |  |  |  |  |  |
| **E3 ubiquitin-protein ligase Itchy homolog (*itch*)** | -0.85 | 3563 | 1758676-1758700 | Intron 1 | -1.22 | 2 |
|  |  |  |  |  |  |  |
| 4-aminobutyrate aminotransferase, mitocondrial (*abat*) | -0.73 | 2343 | 143576-143600 | Intron 7 | -2.12 | 2 |
|  |  |  |  |  |  |  |
| **Leucine-rich repeat-containing protein 17 (*lrrc17*)** | -0.68 | 3590 | 2397901-2397925 | Prom | -1.80 | 1 |
|  |  |  | 2397926-2397950 | Prom | -1.87 | 1 |
|  |  |  |  |  |  |  |
| Period circadian protein homolog 1 (*per1*) | -1.59 | 3641 | 7974101-7974125 | Intron 7 / Exon 7 | -1.91 | 1 |
|  |  |  |  |  |  |  |
| Ribosomal protein S6 kinase alpha-2 (*rps6ka2*) | -0.90 | 3631 | 3502426-3502450 | Intron 3 | -2.49 | 2 |
|  |  |  |  |  |  |  |
| **Hypoxia-inducible factor 3-alpha (*hif3a*)** | -0.79 | 1579 | 14201-14225 | Intron 1 | -1.76 | 1 |
|  |  |  | 14226-14250 | Intron 1 | -1.76 | 1 |
|  |  |  |  |  |  |  |
| Spermine oxidase (*smox*) | -1.44 | 3595 | 2843126-2843150 | Intron 2 | -1.25 | 1 |
|  |  |  | 2843151-2843175 | Intron 2 | -1.25 | 1 |
|  |  |  |  |  |  |  |
| Tyrosine-protein kinase HCK (*hck*) | -1.08 | 3171 | 338926-338926 | Intron 10 | -1.58 | 1 |
|  |  |  | 339001-339025 | Intron 10 | -0.94 | 1 |
|  |  |  |  |  |  |  |
| **Zinc finger protein 385A-like isoform X2 (*znf385a*)** | -1.05 | 3609 | 2751-2775 | Intron 1 | -1.88 | 2 |
|  |  |  |  |  |  |  |
| **Receptor-type tyrosine-protein phosphatase S (*ptprs*)** | -0.91 | 2897 | 55751-55775 | Prom | -1.71 | 1 |
|  |  |  | 55826-55850 | Prom | -1.31 | 1 |
|  |  |  | 55851-55875 | Prom | -1.31 | 1 |
|  |  |  | 55876-55900 | Prom | -1.31 | 1 |
|  |  |  |  |  |  |  |
| **Solute carrier family 25 member 33 (*slc25a33*)** | -0.79 | 3618 | 179576-179600 | Prom | -1.50 | 2 |
|  |  |  | 179601-179625 | Prom | -1.76 | 2 |
|  |  |  |  |  |  |  |
| **Guanine nucleotide-binding protein G(q) subunit Alpha (*gnaq*)** | -0.73 | 1515 | 21251-21275 | Intron 1 | -1.14 | 1 |
|  |  |  | 21276-21300 | Intron 1 | -1.07 | 1 |
|  |  |  | 21601-21625 | Intron 1 | -1.17 | 1 |
|  |  |  |  |  |  |  |
| **Collagen alpha-6(IV) chain (*col4a6*)** | -0.70 | 2987 | 5301-5325 | Exon 1 | -1.64 | 1 |
|  |  |  | 5326-5350 | Exon 1 | -1.64 | 1 |
|  |  |  |  |  |  |  |
| **Obscurin (*obscn*)** | -1.41 | 3641 | 3312126-3312175 | Intron 1 | -1.10 | 2 |
|  |  |  |  |  |  |  |
| **Calponin-1 (*cnn1*)** | -0.78 | 3408 | 914801-914825 | Prom | -1.11 | 1 |
|  |  |  |  |  |  |  |
| **Cell adhesion molecule-related/down-regulated by oncogenes (*boc*)** | -0.73 | 3612 | 358076-358100 | Intron 1 | -1.54 | 1 |
|  |  |  | 358101-358125 | Intron 1 | -1.54 | 2 |
|  |  |  | 358226-358250 | Intron 1 | -1.56 | 2 |
|  |  |  | 367701-367725 | Intron 1 | -1.65 | 1 |
|  |  |  | 367726-367750 | Intron 1 | -1.65 | 2 |
|  |  |  |  |  |  |  |
| Adipocyte enhancer-binding protein 1 (*aebp1*) | -0.59 | 3044 | 146426-146450 | Intron 19 | -2.07 | 1 |
|  |  |  | 146526-146550 | Intron 19 | -2.07 | 1 |
|  |  |  |  |  |  |  |
| **E3 ubiquitin-protein ligase MYLIP-A (*mylip*)** | -4.13 | 3290 | 2451-2475 | Intron 1 | -1.60 | 1 |
|  |  |  |  |  |  |  |
| TNF receptor-associated factor 4 (*traf4*) | -1.13 | 3366 | 167726-167750 | Exon 7 | -1.46 | 3 |
|  |  |  | 167751-167775 | Exon 7 | -1.05 | 1 |
|  |  |  |  |  |  |  |
| Collagen alpha-6(VI) chain (*col6a6*) | -1.10 | 2913 | 286226-286250 | Intron 25 | -1.24 | 1 |
|  |  |  | 286351-286375 | Intron 25 | -1.13 | 1 |
|  |  |  | 286401-286425 | Intron 25 | -1.06 | 1 |
|  |  |  |  |  |  |  |
| **Collagen alpha-1(XXVIII) chain (*col28a1*)** | -1.09 | 2631 | 103826-103850 | Prom | -1.26 | 2 |
|  |  |  |  |  |  |  |
| **2-hydroxyacylsphingosine 1-beta-galactosyltransferase (*ugt8*)** | -1.02 | 3605 | 2649226-2649250 | Intron 1 | -0.92 | 1 |
|  |  |  | 2649251-2649275 | Intron 1 | -0.92 | 1 |
|  |  |  | 2649301-2649325 | Intron 1 | -1.01 | 1 |
|  |  |  |  |  |  |  |
| **Electroneutral sodium bicarbonate exchanger 1 (*slc4a8*)** | -1.00 | 2788 | 111251-111275 | Intron 1 | -1.70 | 1 |
|  |  |  |  |  |  |  |
| adhesion G-protein coupled receptor D2-like (*adgrd2*) | -0.96 | 3603 | 2050901-2050925 | Intron 4 | -1.54 | 1 |
|  |  |  | 2051526-2051550 | Intron 3 | -1.20 | 1 |
|  |  |  |  |  |  |  |
| collagen alpha-1(XXI) chain-like (*col21a1*) | -0.90 | 3616 | 195901-195925 | Intron 2 | -1.08 | 1 |
|  |  |  | 195926-195950 | Intron 2 | -1.08 | 1 |
|  |  |  | 195976-196000 | Intron 2 | -1.25 | 1 |
|  |  |  |  |  |  |  |
| EGF, latrophilin seven transmembrane domain-containing protein 1 (*adgrl4*) | -0.90 | 3408 | 744176-744200 | Intron 2 | -1.09 | 1 |
|  |  |  | 744301-744325 | Intron 2 | -1.58 | 1 |
|  |  |  | 744326-744350 | Intron 2 | -1.87 | 1 |
|  |  |  |  |  |  |  |
| **G protein-coupled receptor kinase 5 (*grk5*)** | -0.87 | 3361 | 261226-261250 | Intron 1 | -2.37 | 1 |
|  |  |  |  |  |  |  |
| DNA mismatch repair protein (*msh3*) | -0.84 | 3283 | 311751-311775 | Intron 22 | -2.81 | 2 |
|  |  |  | 320226-320250 | Intron 20 | -0.91 | 1 |
|  |  |  | 329601-329625 | Intron 19 | -1.13 | 1 |
|  |  |  |  |  |  |  |
| **Glycerophosphodiester phosphodiesterase 1 (*gde1*)** | -0.82 | 3579 | 1579301-1579325 | Prom | -1.46 | 1 |
|  |  |  |  |  |  |  |
| **Hemicentin-1 (*hmcn1*)** | -0.80 | 3207 | 377676-377700 | Intron 1 | -0.96 | 1 |
|  |  |  |  |  |  |  |
| **Complement component C1q receptor (*cd93*)** | -0.74 | 3466 | 1006376-1006400 | Prom | -1.29 | 1 |
|  |  |  |  |  |  |  |
| EMILIN-2-like (*emilin2*) | -0.72 | 3524 | 1568376-1568400 | Intron 3 | -2.34 | 1 |
|  |  |  | 1568401-1568425 | Intron 3 | -2.34 | 1 |
|  |  |  | 1568501-1568525 | Exon 4 | -1.69 | 2 |
|  |  |  | 1568526-1568550 | Exon 4 | -1.69 | 2 |
|  |  |  | 1568551-1568575 | Exon 4 | -1.45 | 1 |
|  |  |  | 1568576-1568600 | Exon 4 | -1.45 | 1 |
|  |  |  | 1568601-1568625 | Exon 4 / Intron 4 | -1.38 | 2 |
|  |  |  | 1568626-1568650 | Intron 4 | -1.38 | 1 |
|  |  |  |  |  |  |  |
| **Neutral amino acid transporter B (0) (*slc1a5*)** | -0.64 | 3552 | 133826-133850 | Intron 1 | -1.13 | 1 |
|  |  |  |  |  |  |  |
| Carboxypeptidase N catalytic chain (*cpn1*) | -0.64 | 3591 | 1781751-1781775 | Intron 5 | -0.73 | 2 |
